# Effect of atractylenolide III on zearalenone-induced Snail1-mediated epithelial–mesenchymal transition in porcine intestinal epithelium

**DOI:** 10.1186/s40104-024-01038-z

**Published:** 2024-06-07

**Authors:** Na Yeon Kim, Myoung Ok Kim, Sangsu Shin, Woo-Sung Kwon, Bomi Kim, Joon Yeop Lee, Sang In Lee

**Affiliations:** 1 Department of Animal Science and Biotechnology, Kyungpook National University, Sangju, Gyeong-sangbuk-do, 37224 Republic of Korea; 2 Research Institute for Innovative Animal Science, Kyungpook National University, Sangju, Gyeongsangbuk-do 37224 Republic of Korea; 3 National Institute for Korean Medicine Development, Gyeongsan, 38540 Republic of Korea

**Keywords:** Atractylenolide III, Epithelial–mesenchymal transition, IPEC-J2 cells, Snail, TGF-beta signaling, Zearalenone

## Abstract

**Background:**

The intestinal epithelium performs essential physiological functions, such as nutrient absorption, and acts as a barrier to prevent the entry of harmful substances. Mycotoxins are prevalent contaminants found in animal feed that exert harmful effects on the health of livestock. Zearalenone (ZEA) is produced by the *Fusarium* genus and induces gastrointestinal dysfunction and disrupts the health and immune system of animals. Here, we evaluated the molecular mechanisms that regulate the effects of ZEA on the porcine intestinal epithelium.

**Results:**

Treatment of IPEC-J2 cells with ZEA decreased the expression of E-cadherin and increased the expression of Snai1 and Vimentin, which induced Snail1-mediated epithelial-to-mesenchymal transition (EMT). In addition, ZEA induces Snail-mediated EMT through the activation of TGF-β signaling. The treatment of IPEC-J2 cells with atractylenolide III, which were exposed to ZEA, alleviated EMT.

**Conclusions:**

Our findings provide insights into the molecular mechanisms of ZEA toxicity in porcine intestinal epithelial cells and ways to mitigate it.

**Supplementary Information:**

The online version contains supplementary material available at 10.1186/s40104-024-01038-z.

## Background

The intestinal epithelium facilitates essential physiological processes, including food intake, digestion, nutrient absorption, immune response, and waste elimination [1]. In addition, it serves as a critical barrier that prevents the entry of harmful substances such as pathogens, toxins, and foreign antigens, highlighting its importance [2]. However, these harmful substances can affect the intestinal epithelium’s integrity [3]. Damage to the intestinal epithelium can threaten the health of animals [4]. Therefore, maintaining the function of the intestinal epithelium is essential for the physiological balance of the host [5].

Epithelial-to-mesenchymal transition (EMT) is a cellular transdifferentiation process that contributes to tissue remodeling under various pathological conditions, including when the epithelium is damaged [6, 7]. This mesenchymal phenotype is characterized by the acquisition of an elongated fibroblast-like morphology, loss of apicobasal polarity, enhanced cell motility and invasive capabilities, and downregulation of epithelial marker expression, such as that of E-cadherin [8, 9]. Previous research has shown that EMT can occur in the intestinal epithelium when exposed to inflammation and factors such as mycotoxins [10, 11]. Therefore, we hypothesized that exposure of the intestinal epithelium to mycotoxins may induce the transition to mesenchymal cells via EMT during the wound healing process, which could result in impaired intestinal epithelial function.

Zearalenone (ZEA) is a nonsteroidal estrogenic mycotoxin produced by fungi of the *Fusarium* genus that predominantly contaminates staple grains used in animal feed, such as maize, wheat, sorghum, barley, and oats [12, 13]. ZEA is rapidly and extensively absorbed in the intestinal epithelium, potentially leading to gastrointestinal dysfunction, health impairment, immune system dysfunction, and growth delay [14, 15]. Methods to alleviate mycotoxins, including ZEA, involve using a mycotoxin binder (MTB) [16]. However, these MTBs also have the disadvantage of adsorbing organic compounds such as fatty acids, amines, amino acids, vitamins, and aromatic compounds, which share molecular structures, sizes, or surface charges that are similar to those of mycotoxins [17, 18]. Therefore, to minimize the influence of mycotoxins on animals, other products capable of alleviating mycotoxins are needed.

Atractylenolide III (ATL-III) is the main bioactive compound found in *Atractylodes macrocephala* and is a natural product that can be found in medicinal plants such as *Codonopsis* and cocklebur [19]. ATL-III exhibits various pharmacological activities, including anti-inflammatory, anti-allergic, gastrointestinal, and neuroprotective effects [20, 21]. Furthermore, previous studies have reported the alleviating effects of ATL-III on fibrosis, including EMT, in renal fibroblasts and mouse intestinal cells [22, 23]. Therefore, ATL-III is anticipated to be a natural product capable of alleviating EMT in porcine intestinal epithelial cells.

In this study, we aimed to evaluate the impact of ZEA on IPEC-J2 and neonatal porcine jejunum-derived intestinal epithelial cells through gene expression profiling. Furthermore, we sought to identify natural products capable of alleviating the side effects of ZEA and confirm their efficacy.

## Methods

### Cell culture and treatment

IPEC-J2 cells (DSMZ, Braunschweig, Germany) were isolated from the jejunal epithelium of unbuckling piglets. They were cultured in Dulbecco’s Modified Eagle Medium (Thermo Fisher Scientific, Wilmington, DE, USA) supplemented with 10% fetal bovine serum and 1% penicillin-streptomycin. The cells were maintained at 37 °C in a CO_2_ incubator. ZEA (Sigma-Aldrich, St. Louis, MO, USA) was prepared for the treatments by dilution with dimethyl sulfoxide (DMSO) before application to the IPEC-J2 cells.

### Cell viability

IPEC-J2 cells were plated at a density of 3 × 10^4^ cells/well in a 96-well plate. They were cultured for 24 h and subsequently treated with ZEA at concentrations of 0, 5, 10, 20, 40, and 80 μg/mL for 48 h [24]. The cells were then incubated for 2 h with water-soluble tetrazolium 1 (WST-1) (Roche Diagnostics GmbH, Mannheim, Germany). Cell viability was determined by measuring the absorbance of the dye, with background levels subtracted, at the wavelength range of 450–600 nm. This analysis was performed using the GloMax Discover Multi-Microplate Reader. The obtained absorbance values were converted to percentages and subsequently compared with the control group for the assessment of cell viability.

### Gene-expression profiling

IPEC-J2 cells were treated with ZEA for 48 h before RNA extraction. Total RNA was isolated using the AccuPrep Universal RNA Extraction Kit, and its quality was assessed using an Agilent 2100 bioanalyzer with an RNA 6000 Nano Chip (Agilent Technologies, Amstelveen, Netherlands). RNA quantification was performed using a NanoDrop-2000 spectrophotometer (Thermo Fisher Scientific). Library construction was performed using the QuantSeq 3′ mRNA-Seq Library Prep Kit (Lexogen, Vienna, Austria) in accordance with the manufacturer’s protocols. In brief, an oligo-dT primer with an Illumina-compatible sequence at its 5′ end was hybridized with RNA (500 ng), followed by reverse transcription. After RNA template degradation, second-strand synthesis was initiated using random primers with an Illumina-compatible linker sequence at the 5′ end. The resulting double-stranded library was purified using magnetic beads to remove the residual reaction components. Furthermore, the library underwent amplification to incorporate complete adapter sequences necessary for cluster generation. The final library was purified from the polymerase chain reaction (PCR) components, and high-throughput sequencing was performed as single-end 75 sequencing using the Next Seq 500 platform (Illumina, San Diego, CA, USA). Following this, the gene-expression data were validated using an Excel-based analysis of differentially expressed genes (DEGs). DEGs were analyzed using Gene Ontology (GO) and Kyoto Encyclopedia of Genes and Genomes (KEGG) mapping via the Database for Annotation, Visualization, and Integrated Discovery.

### RT-PCR

Total RNA was extracted using the AccuPreP Universal RNA Extraction Kit (BioNEER, Daejeon, Korea). cDNA was synthesized from 1 μg of total RNA using the DiaStar™ RT Kit (SolGent, Daejeon, Korea). Primers for the target genes in qPCR were designed using Primer 3 (http://frodo.wi.mit.edu). The RT-qPCR protocol involved incubation at 95 °C for 3 min, followed by 40 cycles of 95 °C for 15 s, 56–58 °C for 15 s, and 72 °C for 15 s. The target gene levels were determined using the 2^-ΔΔCt^ method, with normalization to glyceraldehyde-3-phosphate dehydrogenase (GAPDH). The primer sequences are provided in Supplementary Material 1 (Table S1).

### Immunofluorescence staining of cells

IPEC-J2 cells were subjected to various treatments, including ZEA, TGF-β, TGF-β + siRNA1460, ZEA + TGF-β inhibitor, and ZEA + siRNA1460, and were cultured on gelatin-coated glass coverslips for 24 h. The cells were fixed with 4% paraformaldehyde for 15 min and subsequently treated with a blocking buffer for 1 h. Mouse anti-E-cadherin IgG (Cell Signaling Technology, Danvers, MA, USA) was applied in a 1:200 antibody solution and incubated overnight. Following three 3-min washes with phosphate-buffered saline, goat antimouse secondary antibody (Abcam, Cambridge, UK) was applied at a 1:500 ratio and incubated for 1 h in the dark. The nuclei were counterstained with DAPI (Vector Laboratories, Burlingame, CA, USA), and the cells were covered with a cover slip. The cells were photographed under a fluorescence microscope (Korealabtech, Seongnam-si, Republic of Korea).

### Gene silencing of Snai1 by small interfering RNA (siRNA)

The following target sequences were used to inhibit Snai1 expression: SNAI1–444 (5′-GUCCUUCUCUUCCACCUCA-3′), SNAI1–1393 (5′-CUAUUUCAGCCUCCUGUUU-3′), and SNAI1–1460 (5′-GACUGUGAGUAAUUGCCUU-3′). Transfection with siRNA was performed following the manufacturer’s instructions (BioNEER, Daejeon, Korea). IPEC-J2 cells were plated at a 3.0 × 10^4^ cells/well density in a 6-well plate in a growth medium without antibiotics. Transfection was performed using Lipofectamine™ RNAIMAX and siRNA duplexes, followed by 5–6 h incubation at 37 °C. Finally, the medium was replaced with fresh medium containing ZEA and serum for gene knockdown assays after 48 h.

### High-throughput screening (HTS) assay for natural products

For HTS analysis, we were kindly provided 350 natural products from the National Development Institute for Korean Medicine (Gyeongsan, South Korea). All products were diluted to 1 mg/mL using DMSO. IPEC-J2 cells were cultured in 96-well plates at a density of 5 × 10^3^ cells/100 μL for 24 h. The cells were treated with a mixture of ZEA (6.8 μg/mL) and natural products at 20 ng/μL for 48 h. After treatment, 10 μL of EZ-Cytox was added to each mixture, and the reaction proceeded for 2 h. Cell viability was determined by measuring the absorbance of the dye, excluding the background levels, in the wavelength range of 450–600 nm. This analysis was conducted using the GloMax Discover Multi-Microplate Reader.

### Statistical analysis

All experiments were independently conducted in triplicate. Significant differences between treatments were assessed using the General Linear Model (PROC-GLM) procedure within the SAS software. Cell viability data and PCR results were analyzed using the general linear model and *t-*test. Statistical significance was established at *P* < 0.05.

## Results

### Cytotoxicity of ZEA on porcine small intestinal epithelial cells

**Fig. 1:**
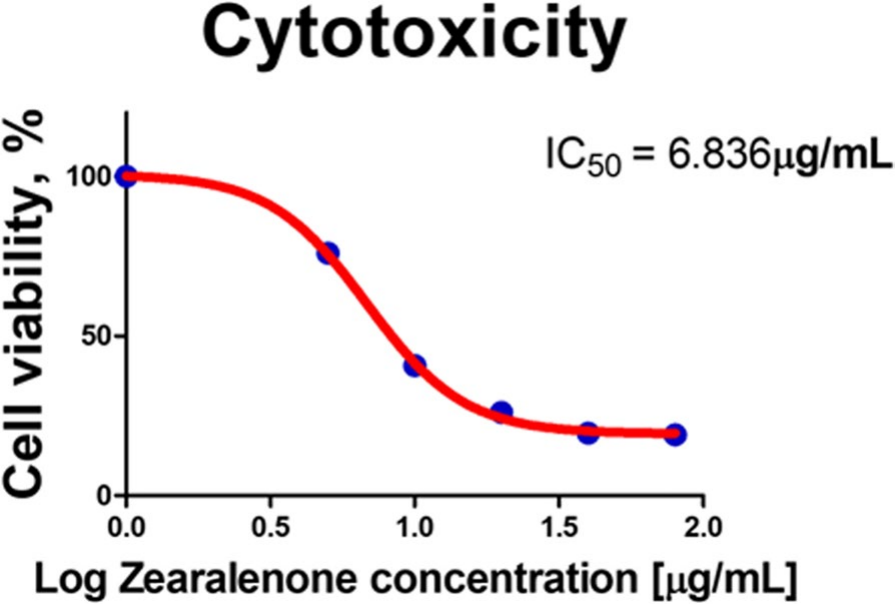
Zearalenone (ZEA) decreased the viability of IPEC-J2 cells. The data present the IC_50_ values after treatment for 48 h with 0, 5, 10, 20, 40, and 80 μg/mL of ZEA in IPEC-J2 cells

To confirm the cytotoxic effect of ZEA on IPEC-J2 cells, cell viability analysis was performed using the WST-1 assay. As the cells were exposed to various concentrations of ZEA (0–80 μg/mL), cell viability decreased in a concentration-dependent manner (Fig. 1). The IC_50_ value of ZEA in IPEC-J2 cells (after 48 h of treatment) was determined to be 6.836 μg/mL. However, we used a 6.8 μg/mL concentration of ZEA for further experiments.

### Identification and validation of DEGs and comparative verification using RNAseq

**Fig. 2:**
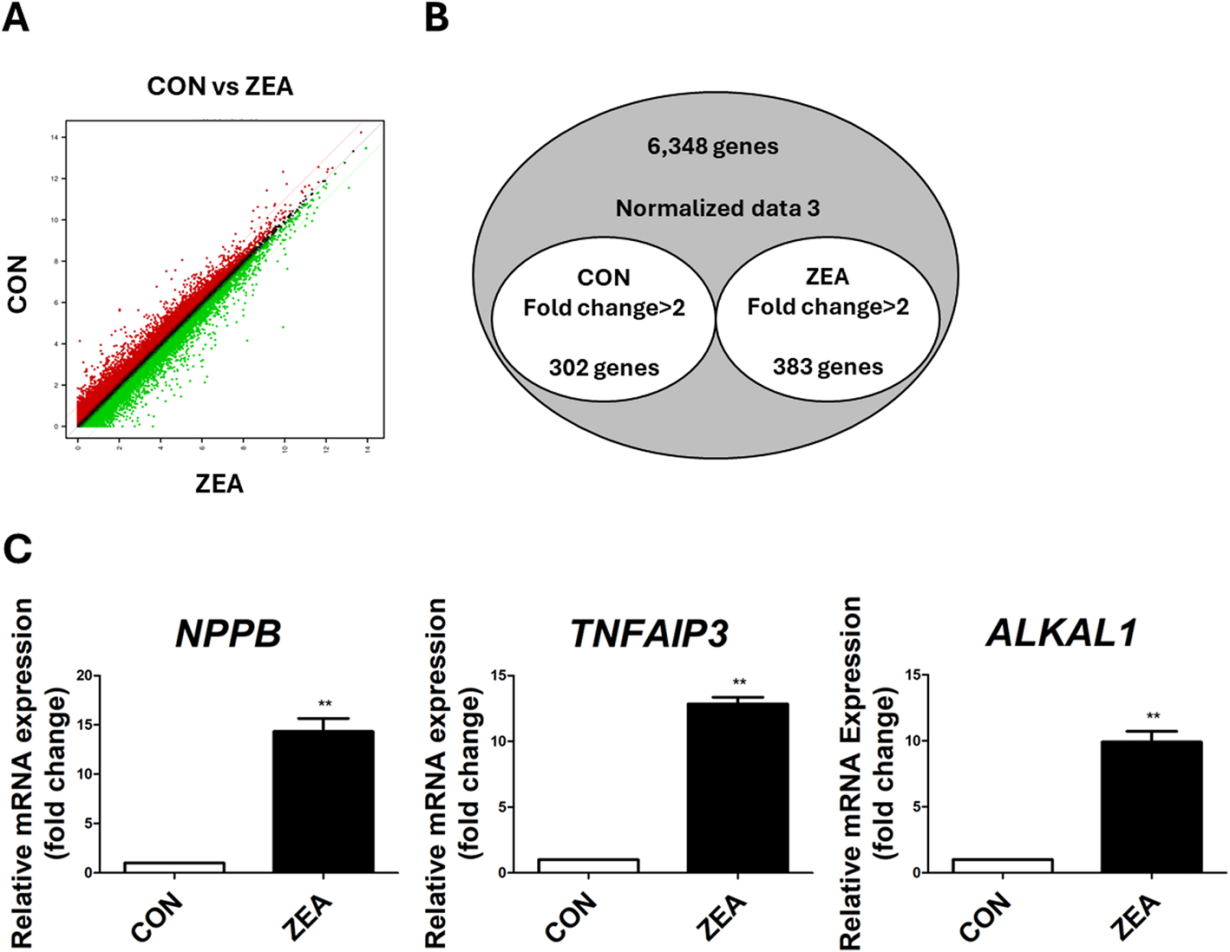
Gene expression profiling of IPEC-J2 cells treated with zearalenone (ZEA) for 48 h. **A** The scatter plot visualizes the gene expression patterns between the control group and the ZEA-treated group. The *x*-axis indicates the normalized data (log2) of the control group, while the y-axis indicates the normalized data (log2) after ZEA treatment. Below the green diagonal line are the genes whose expression decreased at least 2-fold, and above the red diagonal line are the genes whose expression increased at least 2-fold. **B** Venn diagram illustrations of the genes that were differentially expressed after ZEA treatment. A total of 6,348 common genes were upregulated by at least 2-fold (*P* < 0.05). **C** Quantitative expression analysis of highly expressed genes in IPEC-J2 cells after ZEA treatment (*n* = 3). Real-time quantitative PCR analysis was performed in triplicate, and the results were normalized to the expression levels of glyceraldehyde 3-phosphate dehydrogenase (*GAPDH*). Significant differences between the control and treatment groups are indicated by ** (*P* < 0.01). Error bars indicate the standard error (SE) of triplicate analyses

To identify specific DEGs, we performed gene-expression profiling of the small intestinal epithelial cells with or without ZEA treatment (Fig. 2A). Out of the 685 annotated DEGs, 383 were found to be upregulated, and 302 were downregulated (Fig. 2B and Table S2–9). To verify the expression of the DEGs, we analyzed the expression of the top 3 genes through RT-qPCR in the IPEC-J2 cells, treated with or without ZEA. *NPPB* (*P* < 0.01), *TNFAIP3* (*P* < 0.01), and *ALKAL1* (*P* < 0.01) were upregulated in the IPEC-J2 cells after ZEA treatment compared with those without ZEA treatment (Fig. 2C).

### Analysis of GO and KEGG pathways in upregulated and downregulated DEGs

**Fig. 3:**
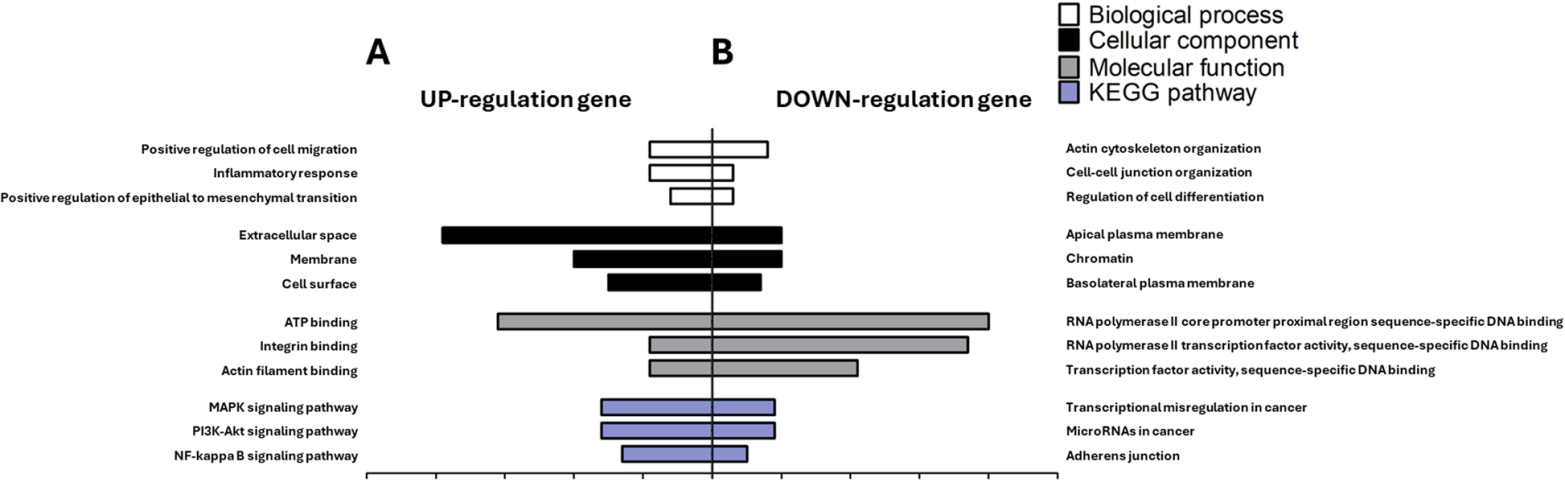
Gene Ontology (GO) and Kyoto Encyclopedia of Genes and Genomes (KEGG) pathway of upregulated differentially expressed genes (DEGs) and downregulated DEGs. **A** The graph includes the GO and KEGG pathway analyses of the upregulated genes. GO is composed of biological processes, cellular components, and molecular functions. **B** The graph includes the GO and KEGG pathway analyses of the downregulated genes

We conducted GO analysis on the 383 upregulated DEGs and identified them related to EMT for biological processes, cellular components, molecular functions, and KEGG pathways. The “biological processes” category of the upregulated DEGs included “Positive regulation of cell migration”, “Inflammatory response” and “Positive regulation of epithelial-to-mesenchymal transition” [25, 26]. The “cellular components” category included “Extracellular space”, “Membrane” and “Cell surface” [27–29]. The “molecular functions” category included “ATP binding”, “Integrin binding” and “Actin filament binding” [30–32]. The “KEGG pathways” category included the “MAPK signaling pathway”, “PI3K-Akt signaling pathway” and “NF-kappa B signaling pathway” [33](Fig. 3A). The “biological processes” category of the downregulated DEGs included “Actin cytoskeleton organization”, “Cell-cell junction organization” and “Regulation of cell differentiation” [34–36]. The “cellular components” category included “Apical plasma membrane”, “Chromatin” and “Basolateral plasma membrane” [37, 38]. The “molecular functions” category included “RNA polymerase II core promoter proximal region sequence-specific DNA binding”, “RNA polymerase II transcription factor activity, sequence-specific DNA binding” and “Transcription factor activity, sequence-specific DNA binding” [39, 40]. The “KEGG pathways” category included “Transcriptional misregulation in cancer”, “MicroRNAs in cancer” and “Adherens junction” [41–43](Fig. 3B).

### Exposure to ZEA induces EMT

**Fig. 4:**
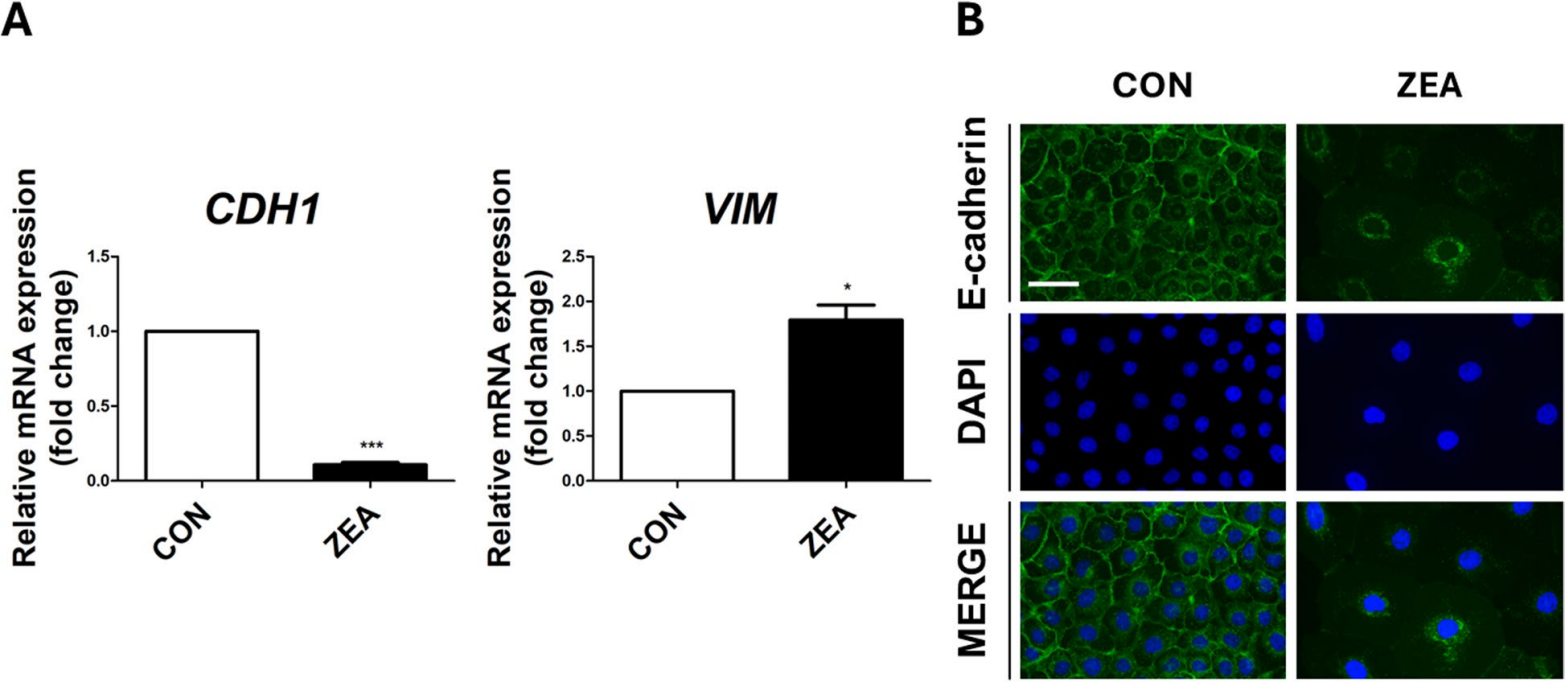
Zearalenone (ZEA) regulates the expression of genes, and E-cadherin is associated with epithelial-to-mesenchymal transition (EMT). **A** Real-time PCR analysis of EMT-related genes such as *CDH1* and *VIM* was conducted in IPEC-J2 cells (*n* = 3). Significant differences between the control and treatment groups are indicated by *** (*P* < 0.001) and * (*P* < 0.05). Error bars indicate the standard error (SE) of triplicate analyses. **B** The immunocytochemical analysis revealed that E-cadherin in IPEC-J2 cells was modulated by ZEA treatment. Immunofluorescence staining for E-cadherin (green) revealed a membranous expression pattern. Nuclei were stained with 4′,6-diamidino-2-phenylindole (DAPI; blue). Scale bar = 40 μm

The GO analysis confirmed the upregulation of EMT. We hypothesized that this would influence the transition of small intestinal epithelial cells to mesenchymal cells when ZEA exposure causes cellular damage. Therefore, we assessed the expression of EMT-related genes and proteins in IPEC-J2 cells using RT-qPCR and immunocytochemistry to determine whether ZEA exposure induced EMT. As expected, the RT-qPCR results showed that IPEC-J2 cells treated with 6.8 μg/mL of ZEA for 48 h led to a decrease in the expression of *CDH1* (*P* < 0.001) and an increase in the expression of *VIM* (*P* < 0.05) (Fig. 4A). Moreover, immunocytochemistry results demonstrated a significant disruption in E-cadherin expression when treated with ZEA for 48 h compared with the control group (Fig. 4B). These results suggest that ZEA induces EMT in the small intestinal epithelial cells.

### TGF-β regulated Snail1-mediated EMT

**Fig. 5:**
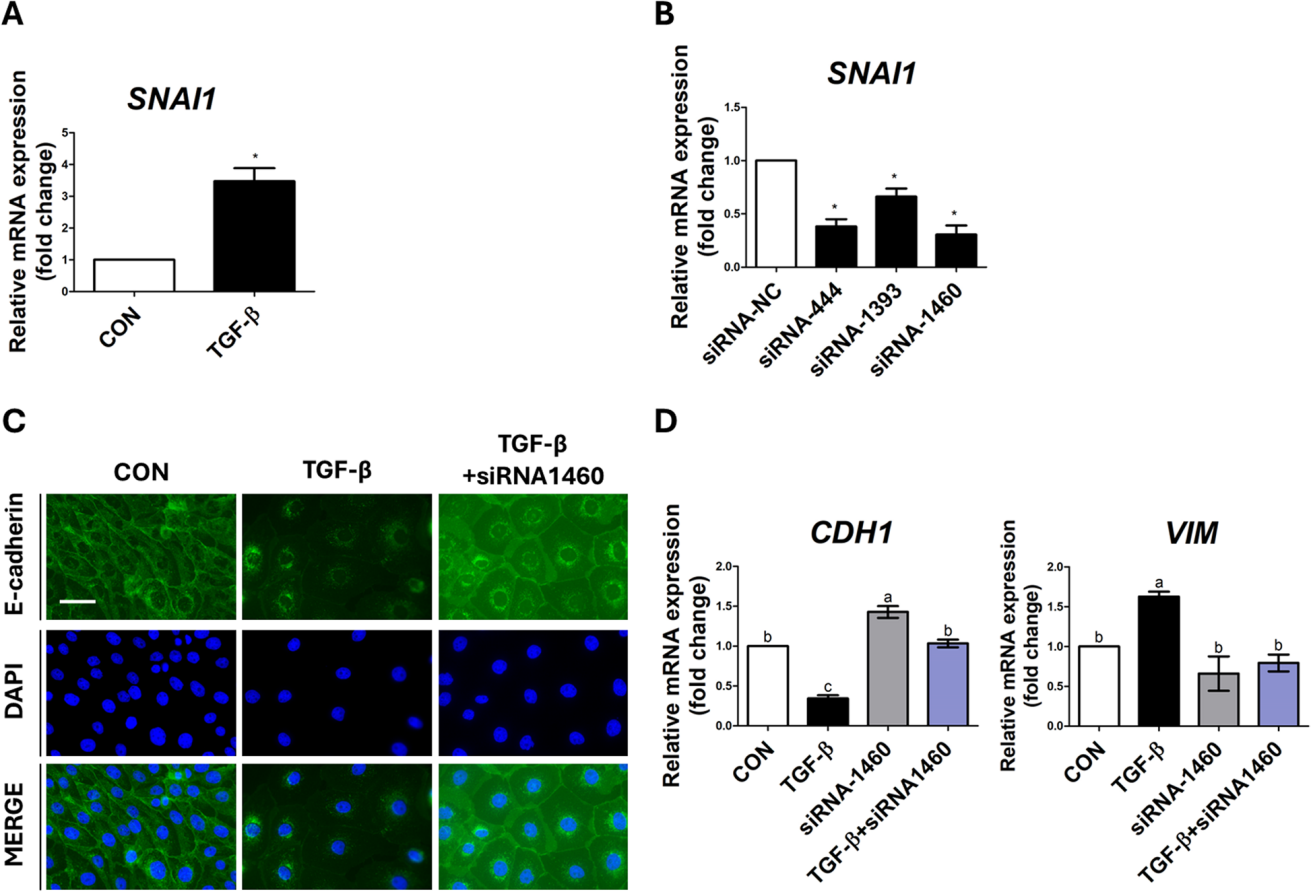
TGF-β induces Snai1-mediated epithelial-to-mesenchymal transition (EMT). **A** The expression of *SNAI1* in IPEC-J2 cells treated with or without TGF-β was analyzed by real-time PCR (*n* = 3). **B** The mRNA expression level of *SNAI1* in IPEC-J2 cells after treatment with siRNA-*SNAI1* was analyzed by real-time PCR (*n* = 3). Significant differences between the control and treatment groups are indicated as * (*P* < 0.05). **C** and **D** Immunofluorescence staining and real-time PCR (*n* = 3) revealed the effects of siRNA-1460 on the expression of EMT-related proteins and genes in TGF-β-treated IPEC-J2 cells. Nuclei were stained with 4′,6-diamidino-2-phenylindole (DAPI; blue). Scale bar = 40 μm. Based on Duncan’s multiple range test, lowercase letters (a, b, and c) indicate significant differences between treatments. Error bars represent the standard error (SE) of triplicate analyses

To investigate the impact of the expression of Snai1 via TGF-β signaling, we compared the expression in IPEC-J2 cells treated with Recombinant Mouse TGF-beta 1 protein with that of the control group. The treatment with Recombinant Mouse TGF-beta 1 Protein significantly increased the expression of *SNAI1* (Fig. 5A). Subsequently, we examined whether the induction of EMT by TGF-β signaling was altered by *SNAI1* knockdown. Three siRNA sequences targeting porcine *SNAI1* were designed. We confirmed the knockdown of *SNAI1* in IPEC-J2 cells by comparing them with control cells that were transfected with a nonspecific siRNA, which had no homology to the porcine sequences (Fig. 5B). The knockdown efficiencies of siRNA-444, siRNA-1393, and siRNA-1460 for *SNAI1* were 61.60% (*P* < 0.05), 33.84% (*P* < 0.05), and 69.14% (*P* < 0.05), respectively. We used SNAI1-siRNA-1460 for further experiments. The suppression of *SNAI1* expression, compared with the treatment of only TGF-β, led to an increase in the reduced E-cadherin expression by TGF-β, which was similar to that of the control group (Fig. 5C). In addition, the expression of *CDH1* increased. The expression of *VIM* decreased (Fig. 5D). These results suggested that TGF-β signaling directly regulates the induction of EMT through the mediation of Snail1.

### EMT was induced via the Snail1-mediated TGF-β signaling in response to ZEA toxicity

**Fig. 6:**
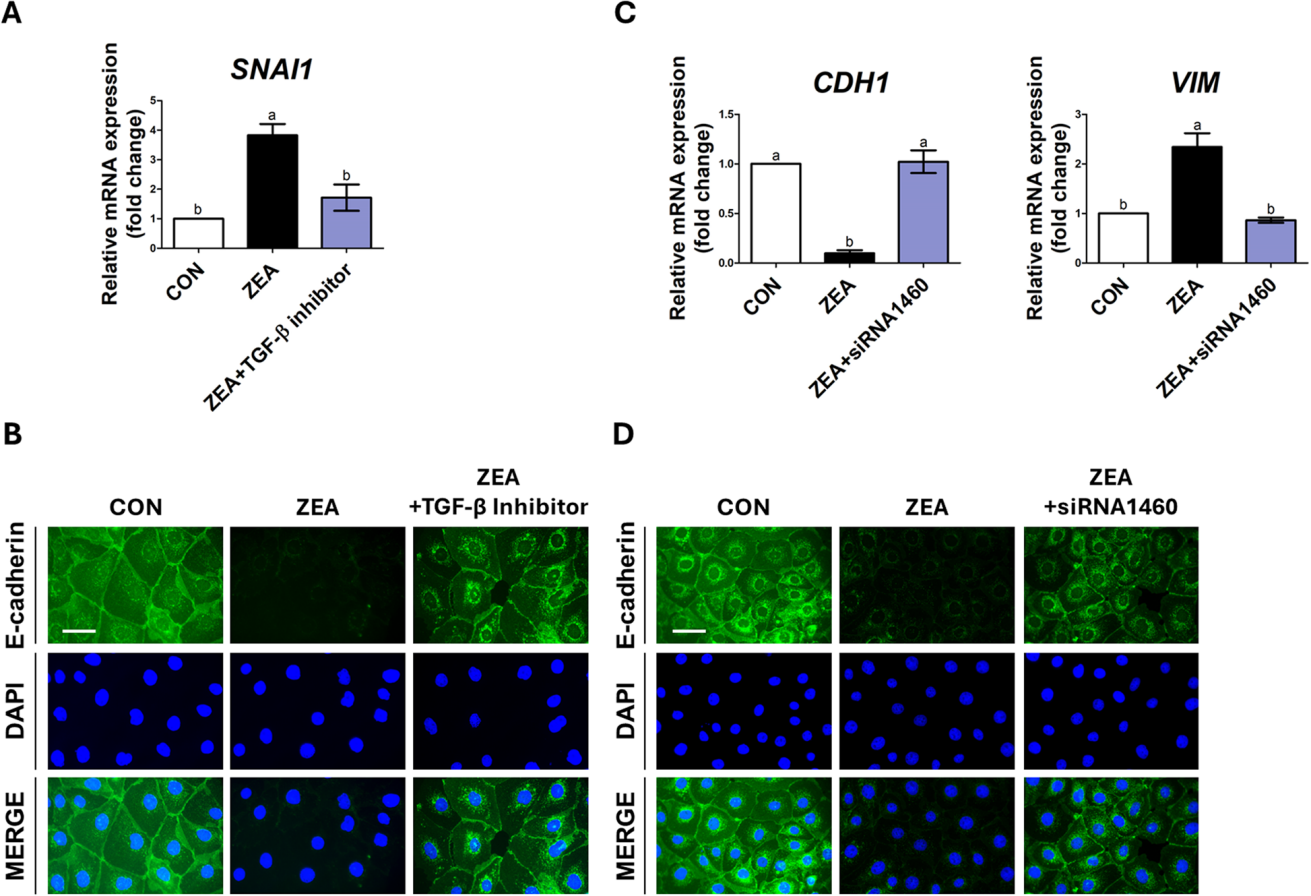
Zearalenone (ZEA) induced Snail-mediated epithelial-to-mesenchymal transition (EMT) via TGF-β signaling. **A** The TGF-β inhibitor decreased the expression of *SNAI1* in ZEA-treated IPEC-J2 cells (*n* = 3). **B** Treatment with a TGF-β inhibitor increased the expression of E-cadherin in ZEA-treated IPEC-J2 cells. **C** and **D** Real-time PCR and immunofluorescence staining revealed the regulation of EMT-related genes and E-cadherin expression after siRNA treatment in ZEA-treated IPEC-J2 cells. Lowercase letters (a and b) indicate significant differences between the treatments based on Duncan’s multiple range test. Error bars represent the standard error (SE) of triplicate analyses. Nuclei were stained with 4′,6-diamidino-2-phenylindole (DAPI; blue). Scale bar = 40 μm

We then verified whether ZEA induced EMT through Snail-mediated mechanisms via the TGF-β signaling pathway. Treatment of the IPEC-J2 cells with ZEA increased the *SNAI1* expression, and inhibition of TGF-β by a TGF-β inhibitor (LY-364947) decreased the *SNAI1* expression as much as that of the control group (Fig. 6A). We further confirmed whether TGF-β signaling affected the reduced expression of E-cadherin after ZEA treatment. Cotreatment with ZEA and a TGF-β inhibitor increased the E-cadherin expression in the IPEC-J2 cells, which had been reduced by ZEA, to the level of the control group (Fig. 6B). Subsequently, we investigated whether ZEA treatment affected the induction of Snail1-mediated EMT. Following the cotreatment with ZEA and siRNA-1460, the expression of *CDH1*, in which ZEA decreased, was increased to the level of the control group. In addition, the increased expression of *VIM* was reduced to the level of the control group (Fig. 6C). In addition, cotreatment with ZEA and siRNA-1460 restored the reduced expression of E-cadherin in IPEC-J2 cells, which had been reduced by ZEA, to the level in the control group (Fig. 6D). From these results, we conclude that ZEA induces EMT through the mediation of Snail1 via the TGF-β signaling pathway in IPEC-J2 cells.

### HTS to identify candidate natural products to alleviate ZEA toxicity in IPEC-J2 cells

**Fig. 7:**
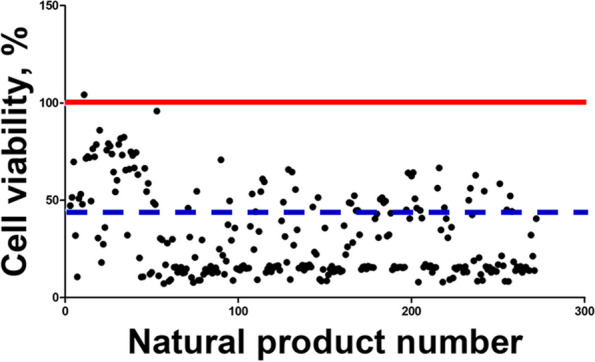
The effect of individual natural products on zearalenone (ZEA) toxicity. IPEC-J2 cells were treated with ZEA at 6.8 μg/mL and each natural product at 20 μg/μL for 48 h. The red line in the graph represents the control group. The blue dashed line in the graph represents the cell viability of cells treated with ZEA alone. The dots represent the cell viability values of the cells treated simultaneously with ZEA and each natural product in the graph

To identify candidate natural products that mitigate the toxicity of ZEA, we screened 270 natural products with only ZEA in IPEC-J2 cells. The number of natural products that exhibited an increase in cell viability, compared with the IC_50_ concentration of ZEA, was 1 with an increase of ≥100%, 1 with an increase of ≥ 80%, 3 with an increase of ≥ 60%, and 16 with an increase of ≥ 20% (Fig. 7). We selected the top 5 candidate natural products among those that increased cell viability as potential natural products that alleviate ZEA-induced EMT toxicity. We have selected ATL-III as the most efficient natural product among them (Fig. S1).

### ATL-III alleviated Snail-mediated EMT induced via TGF-beta signaling induced by ZEA in IPEC-J2 cells

**Fig. 8:**
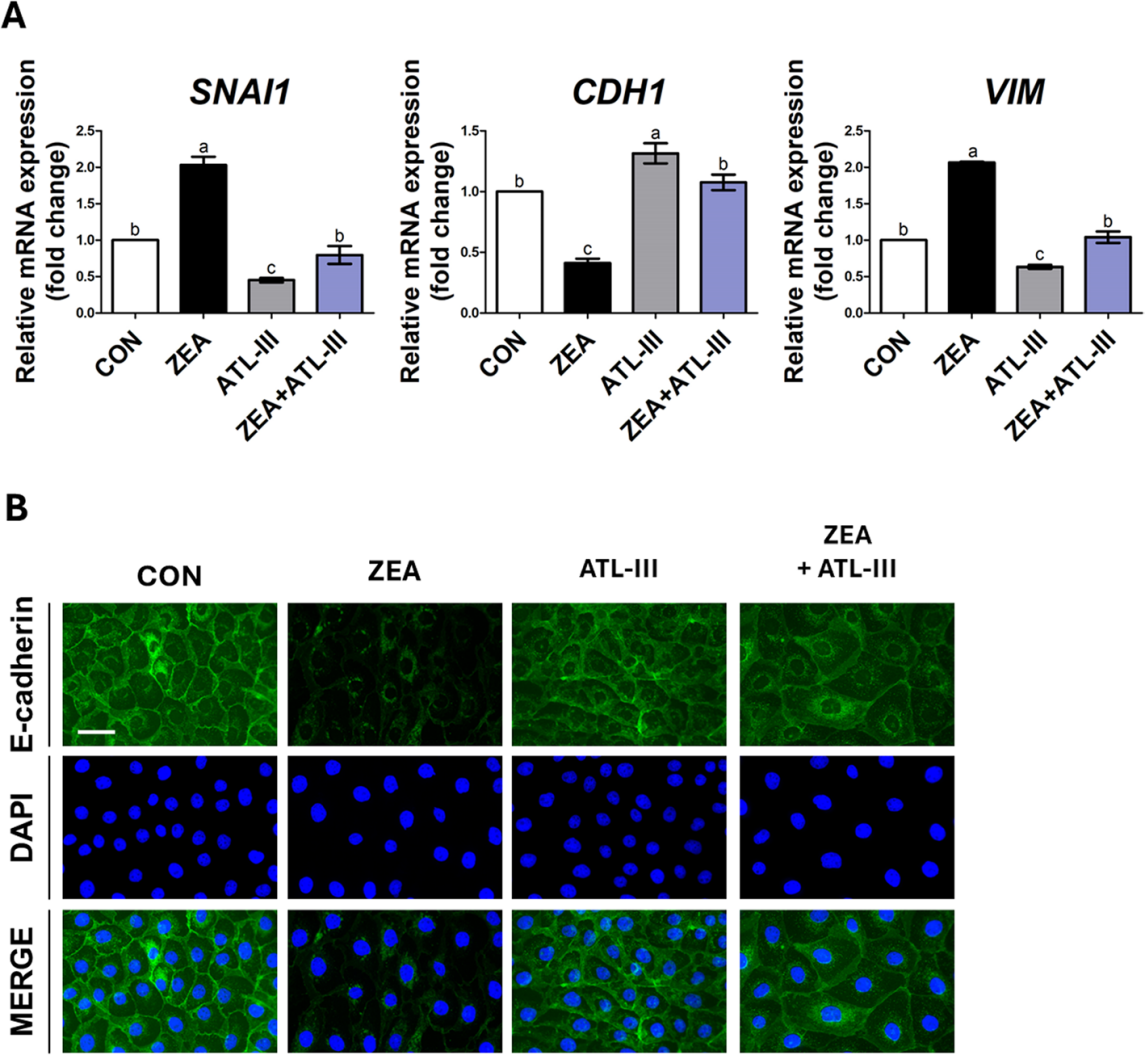
Atractylenolide III (ATL-III) alleviated epithelial–mesenchymal transition (EMT). Real-time PCR analysis of the mRNA expression levels of EMT-related genes in IPEC-J2 cells treated with a combination of ATL-III and zearalenone (ZEA) (*n* = 3). Lowercase letters (a, b, and c) indicate significant differences between the treatments based on Duncan’s multiple range test. Error bars represent the standard error (SE) of triplicate analyses. The expression of E-cadherin under simultaneous treatment with ATL-III and ZEA was confirmed through immunofluorescence staining. Nuclei were stained with 4′,6-diamidino-2-phenylindole (DAPI; blue). Scale bar = 40 μm

We verified whether ATL-III alleviated ZEA-induced EMT. When IPEC-J2 cells were treated with ZEA, the expression of *SNAI1* and *VIM* increased. However, cotreatment with ZEA and ATL-III decreased the expression of *SNAI1* and *VIM* to the level of the control group. After treatment with ZEA, the expression of *CDH1* decreased; however, cotreatment with ZEA and ATL-III increased the expression of *CDH1*, similar to that of the control (Fig. 8A). We also confirmed through immunocytochemistry whether ATL-III influenced the expression of E-cadherin after ZEA treatment. The decreased expression of E-cadherin when treated with ZEA alone was restored when cotreated with ATL-III (Fig. 8B). From these results, it was concluded that ATL-III alleviated EMT induced by Snail via the TGF-β signaling pathway in IPEC-J2 cells.

## Discussion

In livestock production, mycotoxins are significant factors that can detrimentally affect the digestive and immune systems, resulting in growth retardation and increased susceptibility to diseases. This poses a significant problem for livestock production [14, 15]. In particular, mycotoxins primarily target the intestinal epithelium, disrupting normal homeostatic function by affecting intestinal barrier function [44, 45]. Among them, ZEA is one of the most prevalent mycotoxins, posing a severe threat to the health of both animals and humans [46]. Despite several studies demonstrating the toxic effects of ZEA on animals, the molecular mechanisms underlying the impact of ZEA on the intestinal epithelium still require detailed investigation.

Therefore, in the present study, we investigated the gene expression profile of porcine intestinal epithelial cells with or without ZEA treatment. GO analysis revealed an increase in EMT in the biological processes category. EMT occurs as part of the tissue remodeling response when tissues are damaged, which leads to the transition of epithelial cells from an epithelial to a mesenchymal phenotype [47, 48]. During EMT, the expression of epithelial genes such as E-cadherin decreases, whereas mesenchymal genes such as Vimentin increase [49]. Moreover, when EMT is induced, the intestinal epithelium loses function, affecting overall intestinal function and causing impairment [23]. Consistent with previous research findings, our results demonstrate that ZEA induces EMT in IPEC-J2 cells by reducing E-cadherin protein and gene expression, increasing Vimentin gene expression. EMT is characterized by the crucial role of the zinc-finger transcription factor Snail1 as the main inducer [50]. Therefore, we hypothesized that ZEA induces Snail1-mediated EMT. We demonstrated an increase in the expression of the Snail1 and Vimentin genes, along with a decrease in the protein and gene expression of E-cadherin, in IPEC-J2 cells exposed to ZEA. These results suggest that ZEA induces EMT through Snail1 mediation.

In this study, we discovered that ZEA-induced Snail1-mediated EMT in IPEC-J2 cells under ZEA treatment conditions was regulated by TGF-β signaling. The expression of Snail1 is regulated by various factors [51], but Snail mediation via TGF-β signaling is a well-known process in EMT induction [52]. TGF-β signaling regulates various pivotal events of development and physiology and is associated with the onset of diseases such as connective tissue disorders, fibrosis, and cancer [53]. Moreover, TGF-β signaling is highly specialized in epithelial cells and aids in the induction of Snail1-mediated EMT [53, 54]. In contrast, it has been shown that TGF-β signaling inhibition can alleviate these diseases [55]. Therefore, we investigated whether Snail1 in IPEC-J2 cells was impacted by TGF-β signaling. We confirmed that TGF-β treatment significantly increased the expression and induced EMT through EMT-related genes and proteins. We further investigated the relevance of ZEA treatment to Snail1-mediated EMT through TGF-β signaling. Treatment with ZEA significantly increased the expression and experimentally confirmed EMT induction via TGF-β signaling. Therefore, this study demonstrated that ZEA affects Snail1-mediated EMT via TGF-β signaling. This discovery further substantiates the correlation between ZEA and EMT in IPEC-J2 cells.

To overcome the issue of mycotoxins, feed additives containing mycotoxin binders have been utilized [16]. However, the need for alternative solutions has become more apparent because of the drawbacks of mycotoxin binders, which can also absorb vitamins, minerals, and essential nutrients [17, 18]. Natural products are known to have beneficial effects on alleviating mycotoxin contamination [56], and several natural products have been shown to mitigate the toxicity of mycotoxins [57]. Therefore, we focused on identifying natural products that alleviate the EMT induced by ZEA. We screened a pool of natural product candidates, selected specific natural products, and tested their mitigating effects on IPEC-J2 cells. Among the top five natural products identified through HTS, ATL-III has a mitigating impact on the EMT induced by TGF-β signaling [58]. Therefore, we further investigated the effects of ATL-III treatment on Snail1-mediated EMT via TGF-β signaling induced by ZEA in IPEC-J2 cells. We found that ATL-III reduced the expression of Snail1 and alleviated EMT. Consistent with these findings, ATL-III treatment also increased the expression of E-cadherin. Therefore, the findings presented in this study suggest that ATL-III could be used as a feed additive for porcine livestock exposed to ZEA.

## Conclusion

**Fig. 9:**
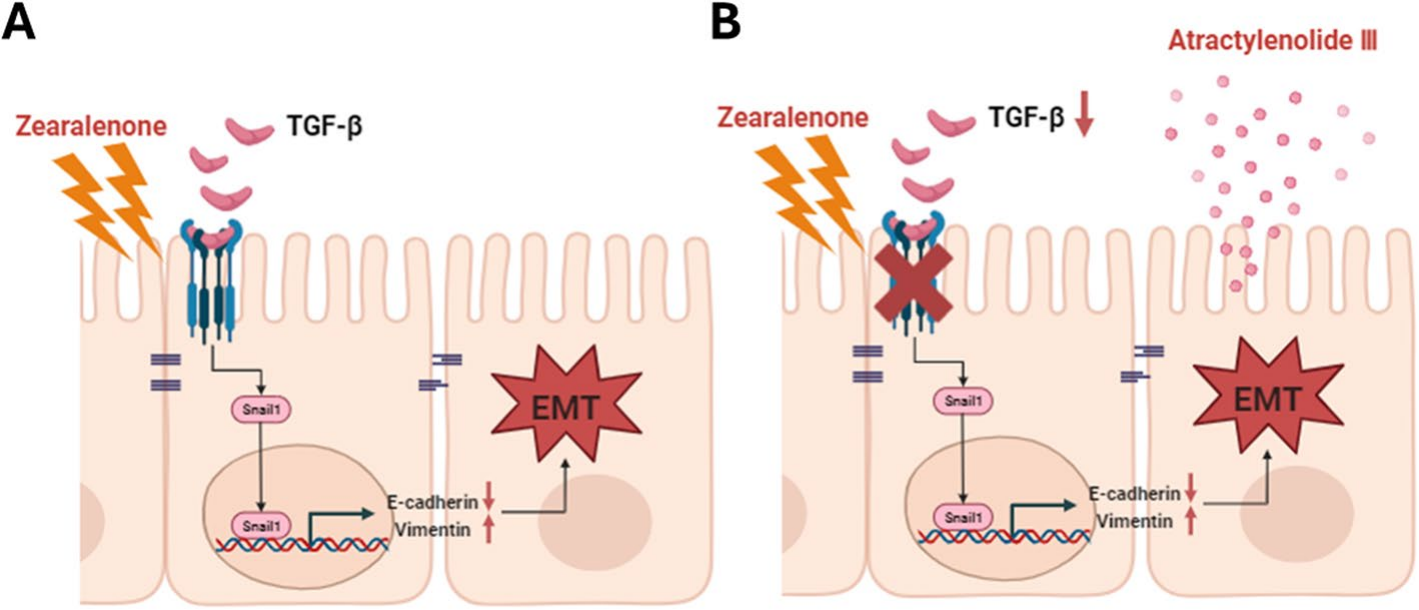
Schematic illustration of the proposed mechanism of the effect of atractylenolide III (ATL-III) on zearalenone (ZEA)-induced epithelial-to-mesenchymal transition (EMT). **A** ZEA induces Snail1-mediated EMT via the TGF-β signaling pathway. **B** ATL-III alleviated ZEA-induced EMT in IPEC-J2 cells

We identified 685 DEGs in porcine intestinal epithelial cells exposed to ZEA using nutritional genomics analysis. We also revealed that ZEA induces EMT by inducing the expression of Snail1 via TGF-β signaling (Fig. 9A). Treatment with ATL-III improved the adverse effects of ZEA on IPEC-J2 cells (Fig. 9B). Collectively, these data indicate that ATL-III could be used as a feed additive for porcine livestock to alleviate ZEA toxicity. This study provides insights into the response of porcine intestinal epithelial cells to ZEA and offers crucial clues for discovering further natural products that can alleviate ZEA toxicity.

### Supplementary Information

**Supplementary Material 1: Fig. S1** The mitigating effects of selected natural substances on epithelial-mesenchymal transition (EMT); **Table S1** List of primers; **Table S2** Biological process in UP-regulation genes (CON vs ZEA); **Table S3** Celluar component in UP-regulation genes (CON vs ZEA); **Table S4** Molecular function in UP-regulation genes (CON vs ZEA); **Table S5** KEGG pathway in UP-regulation genes (CON vs ZEA); **Table S6** Biological process in DOWN-regulation genes (CON vs ZEA); **Table S7** Celluar component in DOWN-regulation genes (CON vs ZEA); **Table S8** Molecular function in DOWN-regulation genes (CON vs ZEA); **Table S9** KEGG pathway in DOWN-regulation genes (CON vs ZEA).

## Data Availability

Not applicable.

## Abbreviations

DEGs: Differentially expressed genes
EMT: Epithelial-to-mesenchymal transition
GO: Gene Ontology
HTS: High-throughput screening
KEGG: Kyoto Encyclopedia of Genes and Genomes
MTB: Mycotoxin-binder
PCR: Polymerase chain reaction
SE: Standard error
ZEA: Zearalenone
