# Supplementary material for: Effect of atractylenolide III on zearalenone-induced Snail1-mediated epithelial–mesenchymal transition in porcine intestinal epithelium
Source: J Anim Sci Biotechnol. 2024 Jun 7;15:80. doi: 10.1186/s40104-024-01038-z (PMC11157892; doi:10.1186/s40104-024-01038-z)
Supplement: Supplementary file 1 — Supplementary Material 1: Fig. S1 The mitigating effects of selected natural substances on epithelial-mesenchymal transition (EMT); Table S1 List of primers; Table S2 Biological process in UP-regulation genes (CON vs ZEA); Table S3 Celluar component in UP-regulation genes (CON vs ZEA); Table S4 Molecular function in UP-regulation genes (CON vs ZEA); Table S5 KEGG pathway in UP-regulation genes (CON vs ZEA); Table S6 Biological process in DOWN-regulation genes (CON vs ZEA); Table S7 Celluar component in DOWN-regulation genes (CON vs ZEA); Table S8 Molecular function in DOWN-regulation genes (CON vs ZEA); Table S9 KEGG pathway in DOWN-regulation genes (CON vs ZEA). [file 40104_2024_1038_MOESM1_ESM.pdf]

Supplementary data

Fig. S1. The mitigating effects of selected natural substances on epithelial-mesenchymal transition (EMT)

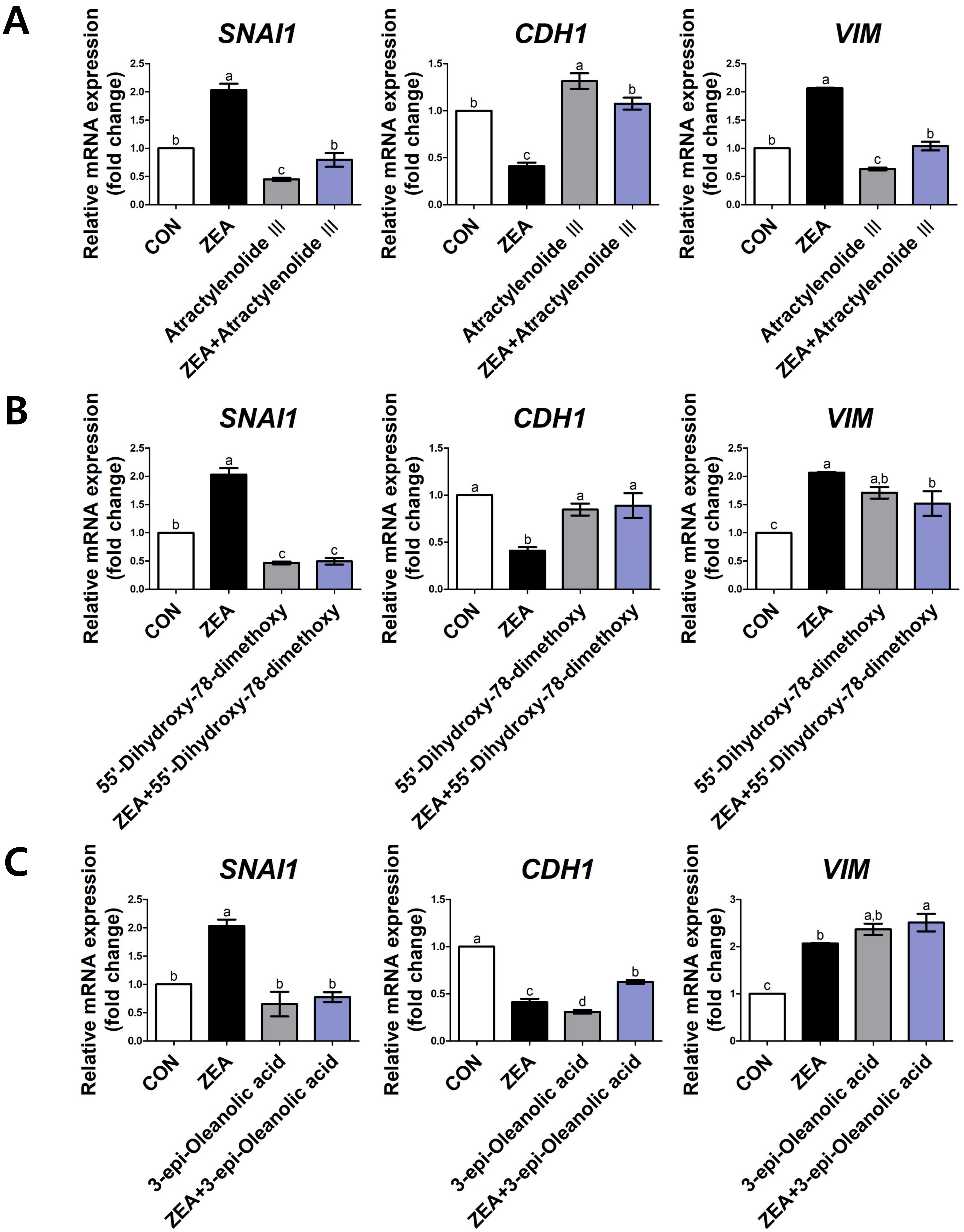

Supplementary data
Table. S1. List of primers

Table 1. List of primers

Genes	Description	Accession No.		Sequence (5'-3')
GAPDH	Glyceraldehyde-3-phosphate dehydrogenase	NM_001206359	Forward	ACACCGAGCATCTCCTGACT
			Reverse	GACGAGGCAGGTCTCCCTAA
SNAI1	Snail family transcriptional repressor 1	XM_021077961.1	Forward	AGGACTCTGAGGACCGTTTC
			Reverse	ACAAACCCACACAGAAAGGC
CDH1	Cadherin 1	NM_001163060.1	Forward	TCTCCTGTTCCTCGACGATG
			Reverse	TGGCCCCATGTGTTAGTTCT
VIM	Vimentin	XM_005668106.3	Forward	ACAAGTCCAAGTTTGCCGAC
			Reverse	CTTCCATTTCCCGCATCTGG
NPPB	Natriuretic peptide B	NM_213846.1	Forward	CAGCAGCCTCTATCCTCTCC
			Reverse	CCTGTATCCCTGGCAGTTCT
TNFAIP3	TNF alpha induced protein 3	XM_005659182.3	Forward	CTTCGGACTTGTCCAGCATG
			Reverse	CCGACATGAAGAAAGGGCAG
ALKAL1	ALK and LTK ligand 1	XM_021089376.1	Forward	TCCTTAACCCACTGAGCGAG
			Reverse	TAGGACACAGCTCACCAGTC

Table. S2. Biological process in UP-regulation genes (CON vs ZEA)

Term	Count	Genes
GO:0007155~cell adhesion	13	SPON1, VCAN, ITGB5, LAMA1, ITGAV, THY1, ITGA5, ATP1B1, CD44, FERMT2, ICAM1, FERMT3
GO:0008284~positive regulation of cell proliferation	11	MARCKSL1, SPDYA, IL18, LIF, ADM, F2, AREG, NKX3-1, EREG, FGFR1, HBEGF
GO:0030335~positive regulation of cell migration	9	EDN1, SEMA7A, ONECUT2, PLAU, PDPN, SNAI1, IRS2, ITGAV, FERMT3
GO:0006954~inflammatory response	9	CSF1, IL23A, AXL, LOC100525396, RARRES2, IL18, REL, CD44, RELB
GO:0008283~cell proliferation	7	USP13, CITED2, TXNRD1, IL18, TACC1, ADM, SDCBP2
GO:0007229~integrin-mediated signaling pathway	7	SEMA7A, ITGB5, ITGAV, THY1, ITGA5, FERMT2, FERMT3
GO:0010718~positive regulation of epithelial to mesenchymal transition	6	BMP4, RGCC, PDPN, SNAI1, FERMT2, LOXL2
GO:0001934~positive regulation of protein phosphorylation	6	SEMA7A, SLCO3A1, RARRES2, PLAUR, F2, NKX3-1
GO:0007507~heart development	6	EDN1, ADAP2, PDLIM2, ADM, PDLIM7, NKX3-1
GO:0051092~positive regulation of NF-kappaB transcription factor activity	6	SLCO3A1, CTH, CAMK2A, IL18, RAB7B, MALT1
GO:0030036~actin cytoskeleton organization	6	PDLIM2, CSRP2, NUAK2, PALLD, DAAM2, PDLIM7
GO:0034446~substrate adhesion-dependent cell spreading	5	AXL, FZD7, ITGAV, FERMT2, FERMT3
GO:0010951~negative regulation of endopeptidase activity	5	SERPINB1, SERPINB2, SERPINE2, SERPINB9, NGF
GO:0001666~response to hypoxia	5	EDN1, PLAU, PLAT, PLOD1, LOXL2
GO:0051897~positive regulation of protein kinase B signaling	5	AXL, IL18, FERMT2, FGFR1, HBEGF
GO:0042474~middle ear morphogenesis	4	EDN1, TSHZ1, SIX1, MSX1
GO:0045737~positive regulation of cyclin-dependent protein serine/threonine kinase activity	4	RGCC, CCND1, SPDYA, CCNYL1
GO:0034620~cellular response to unfolded protein	4	HSP70.2, HSPA8, BAG3, OPTN
GO:0048661~positive regulation of smooth muscle cell proliferation	4	BMP4, EDN1, IL18, HBEGF
GO:0070536~protein K63-linked deubiquitination	4	USP13, CYLD, SPATA2, TNFAIP3

GO:1902042~negative regulation of extrinsic apoptotic signaling pathway via death domain receptors	4	ITPRIP, PEA15, TNFAIP3, ICAM1
GO:0032880~regulation of protein localization	4	ATCAY, MMD, SIX1, NKX3-1
GO:0045666~positive regulation of neuron differentiation	4	BMP4, MMD, BEND6, FGFR1
GO:0001649~osteoblast differentiation	4	BMP4, GDF10, TP53INP2, SNAI1
GO:0006798~polyphosphate catabolic process	3	ATCAY, PRUNE2, PRUNE1
GO:0000185~activation of MAPKKK activity	3	GADD45B, GADD45A, GADD45G
GO:0038061~NIK/NF-kappaB signaling	3	REL, NFKB2, RELB
GO:0090331~negative regulation of platelet aggregation	3	SERPINE2, UBASH3B, SH2B3
GO:0045725~positive regulation of glycogen biosynthetic process	3	DYRK2, IRS1, IRS2
GO:0032967~positive regulation of collagen biosynthetic process	3	BMP4, RGCC, F2
GO:0045741~positive regulation of epidermal growth factor-activated receptor activity	3	AREG, EREG, HBEGF
GO:0060707~trophoblast giant cell differentiation	3	SOCS3, SNAI1, LIF
GO:0007159~leukocyte cell-cell adhesion	3	ITGA5, FERMT3, ICAM1
GO:0042026~protein refolding	3	DNAJB2, HSP70.2, HSPA8
GO:0035335~peptidyl-tyrosine dephosphorylation	3	DUSP5, DUSP10, UBASH3B
GO:0007422~peripheral nervous system development	3	CITED2, NGF, SLC5A3
GO:0033627~cell adhesion mediated by integrin	3	ITGB5, ITGAV, ICAM1
GO:0045840~positive regulation of mitotic nuclear division	3	EDN1, RGCC, EREG
GO:1900745~positive regulation of p38MAPK cascade	3	GADD45B, GADD45A, GADD45G
GO:0042098~T cell proliferation	3	IL23A, CTPS1, MALT1
GO:0035987~endodermal cell differentiation	3	ITGB5, COL12A1, ITGAV
GO:1900182~positive regulation of protein localization to nucleus	3	LIF, F2, FERMT2
GO:0045860~positive regulation of protein kinase activity	3	CSF1, MMD, EREG

GO:0035116~embryonic hindlimb morphogenesis	3	BMP4, WNT7A, MSX1
GO:0034504~protein localization to nucleus	3	BMP4, SIX1, MSX1
GO:0007173~epidermal growth factor receptor signaling pathway	3	AREG, EREG, HBEGF
GO:0051085~chaperone mediated protein folding requiring cofactor	3	HSP70.2, HSPA8, HSPH1
GO:0072193~ureter smooth muscle cell differentiation	2	BMP4, SIX1
GO:0072683~T cell extravasation	2	XG, ICAM1
GO:2000392~regulation of lamellipodium morphogenesis	2	PDPN, CD44
GO:1990108~protein linear deubiquitination	2	CYLD, SPATA2
GO:0060544~regulation of necroptotic process	2	CYLD, SPATA2
GO:0045604~regulation of epidermal cell differentiation	2	KLF7, MAFF
GO:0051454~intracellular pH elevation	2	SLC26A3, SLC26A6
GO:0002291~T cell activation via T cell receptor contact with antigen bound to MHC molecule on antigen presenting cell	2	APBB1IP, ICAM1
GO:0030321~transepithelial chloride transport	2	SLC12A2, SLC26A6
GO:0060536~cartilage morphogenesis	2	SNAI1, MSX1
GO:0070828~heterochromatin organization	2	SNAI1, LOXL2
GO:0002693~positive regulation of cellular extravasation	2	THY1, ICAM1
GO:0010888~negative regulation of lipid storage	2	NFKBIA, ITGAV
GO:0032000~positive regulation of fatty acid beta-oxidation	2	IRS1, IRS2
GO:0060708~spongiotrophoblast differentiation	2	SOCS3, LIF
GO:0051142~positive regulation of NK T cell proliferation	2	IL23A, IL18
GO:0014909~smooth muscle cell migration	2	PLAU, PLAT
GO:0060442~branching involved in prostate gland morphogenesis	2	BMP4, NKX3-1
GO:0035523~protein K29-linked deubiquitination	2	USP13, TNFAIP3
GO:0060054~positive regulation of epithelial cell proliferation involved in wound healing	2	FZD7, WNT7A

Table. S3. Celluar component in UP-regulation genes (CON vs ZEA)

Term	Count	Genes
GO:0005737~cytoplasm	82	DYRK2, SRXN1, ZFAND2A, FBXO25, TNFAIP3, SIX1, ADM, PPP1R18, AREG, NUDT4, NPPB, TUBB6, RGS2, DUSP10, CSRP2, ATCAY, CCND1, UBASH3B, IMPA1, CASP10, CFL2, PEA15, ANKRD1, DUSP5, HSP90AA1, EDN1, RBFOX3, TPM4, FST, IL18, CTPS1, SERPINB9, KCNAB3, SDCBP2, TUBA4A, PDRG1, CLIP4, PEX5, CDC42EP1, FSCN2, OPTN, BRSK1, DNAH8, CAMK2A, HSPA4L, PRUNE2, LHB, PRUNE1, PDXP, GSKIP, SPATA2, METTL21A, RELB, HSPH1, NUAK2, ADAP2, MT-2B, CLMN, BAG3, CREG1, PDE6D, EPS8L3, PDLIM7, HSP70.2, HSPA8, MARCKSL1, SMURF2, GADD45B, GADD45A, TXNRD1, RMDN3, RANBP6, NFKB2, GADD45G, NFKBIA, RGCC, CTH, RCAN2, SCGB1A1, REL, CCNYL1, TACC1
GO:0005829~cytosol	69	CDA, DYRK2, SRXN1, SERPINE2, IRS1, IRS2, HK3, PREX1, RGS2, MAP1LC3A, DUSP10, GM2A, UROD, PEA15, ANKRD1, ITGAV, NINL, NKX3-1, HSP90AA1, WIPI1, SERPINB9, SDCBP2, APBB1IP, PALLD, MAP1B, PEX5, PLIN3, HAP1, TRIM16, ALDH7A1, CD44, OPTN, USP13, PRUNE1, PDXP, METTL21A, MALT1, CAMKK1, RELB, DNAJB2, ARFRP1, HSPH1, BAG3, TP53INP2, PDPN, PDE6D, PIP5K1B, RBM38, HSP70.2, HSPA8, LRRC23, TXNRD1, LIF, CIDEC, NFKB2, NFKBIA, RGP1, DNAJA1, KLF7, CYLD, SPSB1, CASTOR2, P4HA2, SNAI1, SLC25A30, NABP1, NFKBIE, FERMT2, FGFR1
GO:0005615~extracellular space	39	CPM, SEMA7A, SPARC, CSF1, SERPINE2, LAMA1, LHB, COL12A1, PLAT, ADM, AREG, CXCL2, LOXL2, NPPB, PLAU, LIPH, CREG1, GDF10, SERPINB1, EDN1, SERPINB2, FST, LOC100525396, RARRES2, IL18, LIF, WNT7A, SERPINB9, F2, NGF, EREG, BMP4, AXL, IL23A, NPC2, IFNE, SCGB1A1, VWA2, HBEGF

GO:0005576~extracellular region	20	SPON1, MEGF6, PKDCC, RARRES2, PON2, LHB, COL12A1, LIF, PLAT, F2, NGF, BMP4, VCAN, NPC2, LIPH, SCGB1A1, ANOS1, ALKAL1, ST3GAL1
GO:0016020~membrane	20	SEMA7A, MARCKSL1, ITPRIP, ABCC2, SPARC, EPB42, LAMA1, ABO, LOXL2, DNAJA1, XKR8, UGCG, ARFRP1, CCND1, BAG3, COL4A1, REEP2, PEX5, SLC25A30, SERINC4
GO:0009986~cell surface	15	MEGF6, ABCC2, SPARC, ITGB5, PLAUR, PLAT, THY1, ANTXR2, AREG, AXL, PLAU, ANOS1, ITGAV, CD44, HBEGF
GO:0005813~centrosome	12	CYLD, HSP70.2, BRSK1, NIN, RGCC, NME7, SPATC1, HEPACAM2, HAP1, CCDC81, NINL, RELB
GO:0005856~cytoskeleton	10	FRMD6, DYRK2, TPM4, TPM2, EPB41L2, PDE6D, CDC42EP1, TACC1, FSCN2, PPP1R18
GO:0016324~apical plasma membrane	9	SLC12A2, ABCC2, PDPN, SLC39A8, THY1, ATP1B1, SLC26A6, CD44, F2RL2
GO:0030018~Z disc	7	PDLIM2, CSRP2, PALLD, BAG3, CFL2, MYL9, PDLIM7
GO:0005802~trans-Golgi network	7	ARFRP1, CHST1, RGS20, WIPI1, RAB7B, ATP9A, OPTN
GO:0001725~stress fiber	6	PDLIM2, TPM4, PALLD, MYL9, PDLIM7, FERMT2
GO:0001650~fibrillar center	6	TXNRD1, SNAI1, ANKRD1, SPATA2, NFKBIE, MALT1
GO:0005776~autophagosome	5	HSPA8, MAP1LC3A, TP53INP2, HAP1, OPTN
GO:0032587~ruffle membrane	5	PDPN, PDXP, ITGAV, EPS8L3, JCAD
GO:0045171~intercellular bridge	4	EAF1, RMDN3, TEX14, NINL
GO:0031258~lamellipodium membrane	3	PDPN, ITGAV, CD44
GO:0008305~integrin complex	3	ITGB5, ITGAV, ITGA5
GO:0034684~integrin alphav-beta5 complex	2	ITGB5, ITGAV
GO:0098560~cytoplasmic side of late endosome membrane	2	CDIP1, LITAF
GO:0098574~cytoplasmic side of lysosomal membrane	2	CDIP1, LITAF
GO:0030055~cell-substrate junction	2	FERMT2, FERMT3

Table. S4. Molecular function in UP-regulation genes (CON vs ZEA)

Term	Count	Genes
GO:0005524~ATP binding	31	IPPK, BRSK1, RTEL1, PKDCC, DYRK2, SRXN1, DNAH8, CAMK2A, HSPA4L, CAMKK1, HK3, HSPH1, NUAK2, PRKG2, PIP5K1B, MYH10, ATP9A, HSP70.2, HSPA8, HSP90AA1, ABCC2, ENTPD4, CTPS1, TEX14, DNAJA1, NME7, AXL, STK17A, MSH4, ATP13A3, FGFR1
GO:0019901~protein kinase binding	14	SLC12A2, BRSK1, IRS2, TEX14, THY1, ATP1B1, TUBA4A, RELB, CYLD, RGCC, CCND1, SPDYA, CCNYL1, FERMT2
GO:0003779~actin binding	13	MARCKSL1, TPM4, TPM2, PPP1R18, PDLIM2, CLMN, DAAM2, MAP1B, EPB41L2, ANKRD1, EPS8L3, MYH10, PDLIM7
GO:0005102~receptor binding	12	MEGF6, SERPINE2, CADM1, LAMA1, RARRES2, PDPN, PLAUR, PLAT, WIPI1, HAP1, F2, NGF
GO:0003700~transcription factor activity, sequence-specific DNA binding	11	FOSL1, TNFRSF6B, SNAI1, REL, SIX1, ETV5, IKZF4, NFKB2, CREB5, RELB
GO:0005178~integrin binding	9	SEMA7A, ITGB5, TSPAN4, ITGAV, THY1, ITGA5, FERMT2, FERMT3, ICAM1
GO:0051015~actin filament binding	9	TAGLN, MARCKSL1, TPM4, CLMN, TPM2, CFL2, FSCN2, MYH10, FERMT2
GO:0005125~cytokine activity	9	BMP4, GDF10, EDN1, CSF1, IL23A, IFNE, IL18, LIF, WNT7A
GO:0008083~growth factor activity	8	BMP4, GDF10, CSF1, LIF, NGF, AREG, EREG, HBEGF
GO:0051087~chaperone binding	6	USP13, DNAJB2, SLC12A2, HSPA8, BAG3, PDPN
GO:0051082~unfolded protein binding	6	DNAJA1, DNAJB2, HSP70.2, HSPA8, HSP90AA1, PDRG1
GO:0008201~heparin binding	6	BMP4, SERPINE2, LIPH, F2, FGFR1, HBEGF
GO:0031418~L-ascorbic acid binding	4	P4HA2, P4HA3, PLOD1, PAM
GO:0031072~heat shock protein binding	4	HSP70.2, HSPA8, BAG3, PDXP
GO:0030674~protein binding, bridging	4	HSPA8, FSCN2, ATP1B1, OPTN
GO:0019900~kinase binding	4	NIN, GADD45A, SNAI1, TNFAIP3
GO:0004309~exopolyphosphatase activity	3	ATCAY, PRUNE2, PRUNE1
GO:0070064~proline-rich region binding	3	CYLD, CCND1, ITSN1
GO:0004721~phosphoprotein phosphatase activity	3	DUSP5, DUSP10, PDXP
GO:0005154~epidermal growth factor receptor binding	3	AREG, EREG, HBEGF
GO:0004656~procollagen-proline 4-dioxygenase activity	2	P4HA2, P4HA3
GO:0050693~LBD domain binding	2	SPON1, CITED2
GO:0005381~iron ion transmembrane transporter activity	2	SLC25A37, SLC39A8

Table. S5. KEGG pathway in UP-regulation genes (CON vs ZEA)

**Term**	**Count**	**Genes**
ssc05200:Pathways in cancer	23	IFNAR2, HSP90AA1, EDN1, GADD45B, LAMA1, GADD45A, FZD7, TXNRD1, CAMK2A, WNT7A, F2, NFKB2, GADD45G, BMP4, NFKBIA, GSTA4, CCND1, IL23A, COL4A1, PMAIP1, ITGAV, NKX3-1, FGFR1
ssc04010:MAPK signaling pathway	16	DUSP5, HSP70.2, HSPA8, CSF1, GADD45B, GADD45A, NGF, AREG, RELB, EREG, NFKB2, GADD45G, DUSP10, RRAS, LOC100155308, FGFR1
ssc04151:PI3K-Akt signaling pathway	16	IFNAR2, HSP90AA1, CSF1, ITGB5, LAMA1, IRS1, NGF, AREG, EREG, CCND1, CREB3L3, COL4A1, ITGAV, ITGA5, CREB5, FGFR1
ssc05205:Proteoglycans in cancer	14	ITGB5, FZD7, CAMK2A, PLAUR, WNT7A, RRAS, CCND1, PLAU, ITGAV, ITGA5, LOC100155308, CD44, FGFR1, HBEGF
ssc04064:NF-kappa B signaling pathway	13	GADD45B, GADD45A, LOC100525396, TNFAIP3, CXCL2, MALT1, ICAM1, RELB, NFKB2, GADD45G, NFKBIA, CYLD, PLAU
ssc04668:TNF signaling pathway	12	NFKBIA, SOCS3, EDN1, CSF1, CREB3L3, CASP10, LOC100525396, LIF, TNFAIP3, CXCL2, ICAM1, CREB5
ssc05169:Epstein-Barr virus infection	12	NFKBIA, IFNAR2, CCND1, GADD45B, GADD45A, TNFAIP3, NFKBIE, CD44, ICAM1, NFKB2, GADD45G, RELB
ssc04810:Regulation of actin cytoskeleton	12	RGCC, RRAS, ITGB5, CFL2, ITGAV, PIP5K1B, ITGA5, F2, MYH10, LOC100155308, MYL9, FGFR1
ssc04060:Cytokine-cytokine receptor interaction	12	BMP4, GDF10, IFNAR2, TNFRSF6B, CSF1, IL23A, IFNE, LOC100525396, IL18, LIF, NGF, CXCL2
ssc05215:Prostate cancer	10	NFKBIA, HSP90AA1, CCND1, CREB3L3, PLAU, PLAT, ETV5, NKX3-1, FGFR1, CREB5
ssc04022:cGMP-PKG signaling pathway	10	NPPB, RGS2, CREB3L3, IRS1, PRKG2, IRS2, ATP1B1, SLC25A4, MYL9, CREB5
ssc04621:NOD-like receptor signaling pathway	10	NFKBIA, IFNAR2, HSP90AA1, MAP1LC3A, LOC100525396, IL18, TNFAIP3, NLRC4, ANTXR2, CXCL2
ssc04024:cAMP signaling pathway	10	NFKBIA, EDN1, RRAS, CREB3L3, CAMK2A, LHB, ATP1B1, LOC100155308, MYL9, CREB5

ssc05166:Human T-cell leukemia virus 1 infection	10	FOSL1, NFKBIA, CCND1, CREB3L3, MSX1, SLC25A4, ICAM1, NFKB2, CREB5, RELB
ssc05417:Lipid and atherosclerosis	9	NFKBIA, HSP70.2, HSPA8, HSP90AA1, LOC100525396, CAMK2A, IL18, CXCL2, ICAM1
ssc05134:Legionellosis	8	NFKBIA, HSP70.2, HSPA8, LOC100525396, IL18, NLRC4, CXCL2, NFKB2
ssc04610:Complement and coagulation cascades	8	SERPINB2, SERPINE2, PLAU, PLAUR, C3AR1, PLAT, F2, F2RL2
ssc05222:Small cell lung cancer	8	NFKBIA, CCND1, GADD45B, COL4A1, LAMA1, GADD45A, ITGAV, GADD45G
ssc04625:C-type lectin receptor signaling pathway	8	NFKBIA, CYLD, RRAS, IL23A, LOC100155308, MALT1, NFKB2, RELB
ssc04380:Osteoclast differentiation	8	FOSL1, NFKBIA, IFNAR2, SOCS3, CYLD, CSF1, NFKB2, RELB
ssc04210:Apoptosis	8	NFKBIA, GADD45B, CASP10, GADD45A, PMAIP1, NGF, TUBA4A, GADD45G
ssc04140:Autophagy - animal	8	MAP1LC3A, RRAS, IRS1, TP53INP2, IRS2, WIPI1, RAB7B, LOC100155308
ssc05224:Breast cancer	8	CCND1, GADD45B, GADD45A, FZD7, WNT7A, FGFR1, NFKB2, GADD45G
ssc04217:Necroptosis	8	IFNAR2, CYLD, HSP90AA1, CAMK2A, CHMP4C, SPATA2, TNFAIP3, SLC25A4
ssc05225:Hepatocellular carcinoma	8	CCND1, GADD45B, GSTA4, GADD45A, FZD7, TXNRD1, WNT7A, GADD45G
ssc05202:Transcriptional misregulation in cancer	8	GADD45B, GADD45A, PLAU, REL, SIX1, PLAT, ETV5, GADD45G
ssc05167:Kaposi sarcoma-associated herpesvirus infection	8	NFKBIA, IFNAR2, PREX1, MAP1LC3A, CCND1, LOC100525396, CXCL2, ICAM1
ssc04510:Focal adhesion	8	ITGB5, CCND1, COL4A1, LAMA1, ITGAV, PIP5K1B, ITGA5, MYL9
ssc05210:Colorectal cancer	7	CCND1, GADD45B, GADD45A, PMAIP1, AREG, EREG, GADD45G
ssc05410:Hypertrophic cardiomyopathy	7	EDN1, ITGB5, TPM4, LAMA1, TPM2, ITGAV, ITGA5
ssc04068:FoxO signaling pathway	7	CCND1, GADD45B, IRS1, GADD45A, FBXO25, IRS2, GADD45G

ssc05418:Fluid shear stress and atherosclerosis	7	BMP4, HSP90AA1, EDN1, GSTA4, ITGAV, PLAT, ICAM1
ssc04218:Cellular senescence	7	RRAS, CCND1, GADD45B, GADD45A, SLC25A4, LOC100155308, GADD45G
ssc05217:Basal cell carcinoma	6	BMP4, GADD45B, GADD45A, FZD7, WNT7A, GADD45G
ssc04137:Mitophagy - animal	6	MAP1LC3A, RRAS, CITED2, RAB7B, LOC100155308, OPTN
ssc04657:IL-17 signaling pathway	6	FOSL1, NFKBIA, HSP90AA1, LOC100525396, TNFAIP3, CXCL2
ssc04512:ECM-receptor interaction	6	ITGB5, COL4A1, LAMA1, ITGAV, ITGA5, CD44
ssc05323:Rheumatoid arthritis	6	CSF1, IL23A, LOC100525396, IL18, CXCL2, ICAM1
ssc05414:Dilated cardiomyopathy	6	ITGB5, TPM4, LAMA1, TPM2, ITGAV, ITGA5
ssc05146:Amoebiasis	6	COL4A1, LAMA1, LOC100525396, SERPINB9, RAB7B, CXCL2
ssc04931:Insulin resistance	6	NFKBIA, SOCS3, CREB3L3, IRS1, IRS2, CREB5
ssc04722:Neurotrophin signaling pathway	6	NFKBIA, IRS1, CAMK2A, NFKBIE, NGF, SH2B3
ssc05218:Melanoma	5	CCND1, GADD45B, GADD45A, FGFR1, GADD45G
ssc05214:Glioma	5	CCND1, GADD45B, GADD45A, CAMK2A, GADD45G
ssc04920:Adipocytokine signaling pathway	5	NFKBIA, SOCS3, IRS1, IRS2, NFKBIE
ssc04115:p53 signaling pathway	5	CCND1, GADD45B, GADD45A, PMAIP1, GADD45G
ssc05220:Chronic myeloid leukemia	5	NFKBIA, CCND1, GADD45B, GADD45A, GADD45G
ssc05216:Thyroid cancer	4	CCND1, GADD45B, GADD45A, GADD45G
ssc04930:Type II diabetes mellitus	4	SOCS3, HK3, IRS1, IRS2
ssc04978:Mineral absorption	4	MT-2B, ATP1B1, SLC26A3, SLC26A6
ssc00240:Pyrimidine metabolism	4	CDA, NME7, ENTPD4, CTPS1
ssc04923:Regulation of lipolysis in adipocytes	4	IRS1, PRKG2, IRS2, CIDEC

Table. S6. Biological process in DOWN-regulation genes (CON vs ZEA)

Term	Count	Genes
GO:0006357~regulation of transcription from RNA polymerase II promoter	46	LOC110262090, ZNF331, AHR, IKZF2, RAI1, FOXS1, HHEX, MECOM, TRPS1, E2F2, STAT6, ALX1, OTX1, JUNB, TEAD3, E2F8, KLF10, OSR2, LOC100521431, EGR1, TCF7L2, SREBF1, SMAD3, ZNF260, BCL11A, OSR1, FOXJ1, RFX3, FOS, KLF3, MEIS2, FOXP2, RUNX1, FOXP1, TBX2, ETV7, MEIS1, TOX2, TFAP4, ELF3, BCL6, TBL1XR1, FOSB, JDP2, MXD3, ZNF432
GO:0000122~negative regulation of transcription from RNA polymerase II promoter	23	EGR1, WNT10B, OSR2, TCF7L2, SREBF1, RARG, SMAD3, CBX4, OSR1, DNMT3A, IGF2, FOXJ1, CXXC5, KLF3, FOXP2, FOXP1, EFNA1, SOX2, BCL6, TBL1XR1, STAT6, ALX1, ZFPM1
GO:0045944~positive regulation of transcription from RNA polymerase II promoter	23	TCF7L2, SREBF1, RNASEL, RARG, NCOA3, OSR1, IGF2, RFX3, ADRB2, EFCAB7, MEIS2, SENP1, SOX2, PLSCR1, TOX2, MEIS1, TBL1XR1, TET3, ALX1, ZFPM1, TLR3, TEAD3, E2F8
GO:0060271~cilium assembly	9	ARL6, CFAP70, WDR90, IQUB, TEKT1, CCDC96, RFX3, B9D1, KIF27
GO:0007507~heart development	8	NEK8, IFT140, OSR1, FOXJ1, DNAAF4, DRC1, TGFBR2, ZFP36L1
GO:0030036~actin cytoskeleton organization	8	LOC110256054, LOC110258364, DAAM1, BCL6, ELMO1, FOXJ1, CRIP2, MTSS1
GO:0007368~determination of left/right symmetry	7	DYNC2LI1, NEK8, DNAI2, IFT140, FOXJ1, DNAAF4, DRC1
GO:0007411~axon guidance	7	EFNA1, SEMA3C, ARTN, SEMA3D, B3GNT2, EFNA4, IGSF9
GO:0042733~embryonic digit morphogenesis	6	OSR2, INTU, IFT140, OSR1, B9D1, TBX2
GO:0030317~flagellated sperm motility	6	TEKT1, TTC21A, CFAP45, ENO4, CCDC40, ENKUR
GO:0006508~proteolysis	6	CFD, CAPN9, ERAP2, CAPN10, CAPN5, ERAP1
GO:0060021~palate development	5	OSR2, INTU, OSR1, DHRS3, TBX2
GO:0050727~regulation of inflammatory response	5	ANXA1, BCL6, LRRC19, CASP1, PIK3AP1
GO:0006869~lipid transport	5	LOC106510284, FABP6, ABCA4, TSPO, C4BPA
GO:0007219~Notch signaling pathway	5	CDH6, CFD, DTX3L, TGFBR2, TBX2
GO:0060285~cilium-dependent cell motility	4	DNAH6, EFHC1, RFX3, DRC1
GO:0009880~embryonic pattern specification	4	MEIS1, SMAD3, PGAP1, MEIS2
GO:0003341~cilium movement	4	DNAI2, SPEF1, CFAP70, DNAAF4
GO:0002062~chondrocyte differentiation	4	WNT10B, OSR2, OSR1, RUNX1
GO:0048511~rhythmic process	4	MAPK10, FGD4, EGR1, AHR

GO:0030501~positive regulation of bone mineralization	4	WNT10B, OSR2, OSR1, ADRB2
GO:0045599~negative regulation of fat cell differentiation	4	WNT10B, SMAD3, ZFPM1, ZFP36L2
GO:0045444~fat cell differentiation	4	TCF7L2, SREBF1, RNASEL, PSMB8
GO:0001570~vasculogenesis	4	MYO1E, JUNB, TGFBR2, ZFP36L1
GO:0071385~cellular response to glucocorticoid stimulus	3	ANXA1, ZFP36L2, ZFP36L1
GO:0035721~intraciliary retrograde transport	3	DYNC2LI1, IFT140, TTC21A
GO:0071549~cellular response to dexamethasone stimulus	3	TFAP4, DDIT4, FBXO32
GO:1902017~regulation of cilium assembly	3	DYNC2LI1, IFT140, MAPK15
GO:0036158~outer dynein arm assembly	3	DNAI2, LRRC49, DNAAF4
GO:0045216~cell-cell junction organization	3	CLDN6, SMAD3, TJP3
GO:0045736~negative regulation of cyclin-dependent protein serine/threonine kinase activity	3	HHEX, TFAP4, INCA1
GO:0044458~motile cilium assembly	3	INTU, FOXJ1, CCDC40
GO:0021915~neural tube development	3	SEMA3C, INTU, ZFP36L1
GO:0003148~outflow tract septum morphogenesis	3	SEMA3C, TGFBR2, TBX2
GO:0045595~regulation of cell differentiation	3	BCL6, SPRY2, RUNX1
GO:0098838~reduced folate transmembrane transport	2	ABCC5, SLC19A1
GO:0060443~mammary gland morphogenesis	2	STAT6, TGFBR2
GO:0008207~C21-steroid hormone metabolic process	2	AKR1D1, HSD17B10
GO:1990086~lens fiber cell apoptotic process	2	E2F2, TGFBR2
GO:1901991~negative regulation of mitotic cell cycle phase transition	2	ZFP36L2, ZFP36L1
GO:0036023~embryonic skeletal limb joint morphogenesis	2	OSR2, OSR1
GO:1904158~axonemal central apparatus assembly	2	SPEF1, DNAJB13
GO:0003430~growth plate cartilage chondrocyte growth	2	RARG, TGFBR2
GO:0031340~positive regulation of vesicle fusion	2	C2CD5, ANXA1
GO:0035166~post-embryonic hemopoiesis	2	MYO1E, SLC37A4

Table. S7. Celluar component in DOWN-regulation genes (CON vs ZEA)

Term	Count	Genes
GO:0005737~cytoplasm	88	OSCP1, PIWIL4, PUS10, MTMR11, CBLB, DUSP18, AHR, IFI44L, IFIT3, HERC5, LOC110258364, HHEX, CAPN9, KYNU, CAPN5, PALM, CASP1, ROPN1L, CHAC1, CAST, SREBF1, ANXA1, TRPC6, CCDC78, DTX3L, S100A1, DAPK1, LRRC49, DNMT3A, IQCD, NCCRP1, PTGR1, LZTFL1, SHROOM4, MLF1, LOC110256054, TMSB15A, C5H12ORF57, FEZ1, TET3, ELMO1, PSME1, S100A6, S100A4, TLR3, CCDC40, LOC100739741, INTU, PRICKLE2, ASAP3, PPM1H, ARL2, CRIP2, CFAP206, CXXC5, ZFP36L2, ZFP36L1, DYNC2LI1, FGD4, S100A16, SNCG, STAT6, TSNAXIP1, VILL, SLC38A2, NEK8, EGR1, SMAD3, ERAP2, STK11IP, INCA1, ERAP1, MOK, ATP2B2, KLHL24, FBXO32, MAPK15, PSMB8, MAPK10, MYO1E, FABP6, CAPN10, LNX1, SPRY2, B9D1, NEK11, PPIL6, BEX5
GO:0005634~nucleus	87	FANK1, ZNF331, PIWIL4, PUS10, RARG, PANK1, NAB2, HNF4G, HJURP, AHR, IRF2BPL, SOX2, HHEX, HMGN5, MECOM, TRPS1, ZNF385B, TEAD3, TGM2, KLF10, LOC100521431, TLE3, OSR2, SREBF1, ANXA1, TIGD4, NCOA3, OSR1, DNMT3A, MTUS1, DYRK1B, NME3, RFX3, WDR72, DNAAF4, FOS, FOXP2, RUNX1, FOXP1, ETV7, TOX2, ELF3, TET3, S100A6, RARB, S100A4, MNS1, ZFPM1, ZNF432, JDP2, LOC110262090, LOC102159655, PIF1, ADRB2, CXXC5, ZFP36L2, ZFP36L1, RAI1, FOXS1, MCRIP2, S100A16, TNNI2, E2F2, STAT6, OTX1, TPPP, RUNX1T1, E2F8, NEK8, TCF7L2, EGR1, SMAD3, BCL11A, MOK, FOXJ1, FBXO32, MEIS2, MAPK15, PSMB8, TBX2, PAN2, MAPK10, MEIS1, TFAP4, FOSB, SPRY2, NEK11
GO:0005654~nucleoplasm	51	RARG, HJURP, CBLB, DUSP18, HNMT, CDH6, KYNU, TRPS1, PALM, HCFC1R1, SLC16A7, JUNB, CFAP45, TLE3, ANXA1, DTX3L, NCOA3, DNMT3A, VWA5A, DYRK1B, ACSL5, FOS, SHROOM4, SENP1, EPN3, ETV7, PLSCR1, TOX2, ELF3, PSME1, ASAP3, PPM1H, FAAP24, CXXC5, RAI1, TNRC18, STAT6, RUNX1T1, E2F8, TCF7L2, EGR1, SMAD3, ZNF260, BCL11A, FBXO32, KLF3, TFAP4, BCL6, FOSB, TJP3, NEK11

GO:0005739~mitochondrion	22	STPG1, PIF1, CPT1A, MGST1, HJURP, ACSL5, SOD2, IFIT3, ACOXL, ADAM28, TFAP4, BCL2L11, DDAH2, CAPN10, CARD19, KYNU, DDIT4, CASP1, TNRC18, TPPP, COQ8A, TGM2
GO:0031514~motile cilium	13	CFAP61, ANXA1, SPEF1, INTU, IQCD, CFAP206, DYNC2LI1, DAAM1, IQUB, TEKT1, ROPN1L, MNS1, DRC1
GO:0036064~ciliary basal body	13	INTU, IFT140, CFAP70, IQCD, CFAP206, MAPK15, DYNC2LI1, CFAP100, DAAM1, CCDC96, LOC100523736, B9D1, SSX2IP
GO:0005930~axoneme	12	DYNC2LI1, SPEF1, SPAG6, IFT140, EFHC1, CCDC96, DNAJB13, MNS1, CFAP206, DRC1, CFAP45, CCDC40
GO:0005667~transcription factor complex	11	TLE3, RARG, MEIS1, SMAD3, RFX3, E2F2, FOS, JUNB, TEAD3, E2F8, TBX2
GO:0016324~apical plasma membrane	10	MUC1, ABCG5, ANXA1, SLC6A9, SPEF1, UPK2, ABCC5, ADRB2, SHROOM4, SLC19A1
GO:0000785~chromatin	10	TCF7L2, EGR1, RARG, SMAD3, HMGN5, TRPS1, NCOA3, RFX3, STAT6, TGM2
GO:0005929~cilium	7	DYNC2LI1, NEK8, ANXA1, TTC21A, CFAP206, KIF27, CCDC40
GO:0016323~basolateral plasma membrane	7	ANXA1, SLC6A9, SPEF1, ABCC5, PALM, SLC16A12, SLC19A1
GO:0016327~apicolateral plasma membrane	3	CLDN6, PALM, KRT8
GO:0097228~sperm principal piece	3	SPAG6, ENO4, ENKUR
GO:0097546~ciliary base	3	NEK8, C9H11ORF97, MOK
GO:0035976~transcription factor AP-1 complex	2	FOS, JUNB

Table. S8. Molecular function in DOWN-regulation genes (CON vs ZEA)

Term	Count	Genes
GO:0000978~RNA polymerase II core promoter proximal region sequence-specific DNA binding	40	LOC110262090, ZNF331, RARG, IKZF2, SOX2, FOXS1, HHEX, MECOM, E2F2, STAT6, OTX1, JUNB, TEAD3, E2F8, KLF10, EGR1, TCF7L2, SREBF1, SMAD3, ZNF260, BCL11A, KCNIP3, DNMT3A, FOXJ1, RFX3, FOS, KLF3, MEIS2, FOXP2, RUNX1, FOXP1, TBX2, MEIS1, TFAP4, ELF3, BCL6, FOSB, JDP2, MXD3, ZNF432
GO:0000981~RNA polymerase II transcription factor activity, sequence-specific DNA binding	37	LOC110262090, ZNF331, SOX2, FOXS1, HHEX, E2F2, STAT6, ALX1, OTX1, JUNB, TEAD3, E2F8, KLF10, OSR2, LOC100521431, EGR1, TCF7L2, SREBF1, SMAD3, OSR1, FOXJ1, RFX3, FOS, KLF3, MEIS2, FOXP2, RUNX1, FOXP1, TBX2, ETV7, MEIS1, TFAP4, ELF3, FOSB, JDP2, MXD3, ZNF432
GO:0042802~identical protein binding	29	GRB7, NAB2, PPM1H, ILDR1, HJURP, OLFML2A, RASGRP1, IFIT3, CASP1, AGR2, STAT6, ROPN1L, TMEM38B, E2F8, NQO1, VPS9D1, DAPK1, DNMT3A, CIDEB, FOS, LZTFL1, MTSS1, DAAM1, BCL6, LNX1, S100A4, MNS1, PIK3AP1, TLR3
GO:0003700~transcription factor activity, sequence-specific DNA binding	21	TCF7L2, SMAD3, BCL11A, ZNF260, HNF4G, RFX3, AHR, FOS, IKZF2, FOXP2, RUNX1, FOXP1, ETV7, FOXS1, ELF3, MECOM, TRPS1, FOSB, STAT6, JDP2, TEAD3
GO:0001228~transcriptional activator activity, RNA polymerase II transcription regulatory region sequence-specific binding	20	KLF10, EGR1, OSR2, SMAD3, FOXJ1, FOS, MEIS2, PLSCR1, HHEX, MEIS1, TFAP4, ELF3, MECOM, FOSB, E2F2, STAT6, ALX1, OTX1, JUNB, ZNF432
GO:0005509~calcium ion binding	20	C2CD5, ANXA1, S100A2, S100A1, KCNIP3, CETN2, CBLB, EFCAB12, EFCAB7, RASGRP1, THBS3, CDH6, PLSCR1, CAPN9, S100A16, S100A6, EFHC1, CALM3, S100A4, TGM2
GO:0001227~transcriptional repressor activity, RNA polymerase II transcription regulatory region sequence-specific binding	12	ETV7, HHEX, FOXS1, BCL6, BCL11A, KCNIP3, TRPS1, MXD3, FOXP2, FOXP1, E2F8, TBX2
GO:0043565~sequence-specific DNA binding	9	ETV7, FOXS1, RARG, ELF3, TRPS1, HNF4G, RARB, FOXP2, FOXP1
GO:0022857~transmembrane transporter activity	8	OSCP1, SLC46A3, SLC7A4, SLC22A23, LOC100626318, IQUB, SLC16A12, SLC37A4
GO:0000977~RNA polymerase II regulatory region sequence-specific DNA binding	8	ETV7, OSR2, LOC100521431, TRPS1, OSR1, ALX1, KLF3, MXD3
GO:0048306~calcium-dependent protein binding	7	ANXA1, S100A2, S100A16, S100A1, S100A6, CALM3, S100A4
GO:0004198~calcium-dependent cysteine-type endopeptidase activity	4	ADGB, CAPN9, CAPN10, CAPN5
GO:0004879~RNA polymerase II transcription factor activity, ligand-activated sequence-specific DNA binding	4	SREBF1, RARG, RARB, AHR
GO:0045504~dynein heavy chain binding	3	DYNC2LI1, DNAI2, LRRC49
GO:0046914~transition metal ion binding	3	S100A2, S100A16, S100A4
GO:0030215~semaphorin receptor binding	3	SEMA3C, SEMA3D, SEMA3B

Table. S9. KEGG pathway in DOWN-regulation genes (CON vs ZEA)

Term	Count	Genes
ssc05200:Pathways in cancer	21	NQO1, WNT10B, TCF7L2, SMAD3, DAPK1, NCOA3, IGF2, MGST1, FOS, RASGRP1, RUNX1, TGFBR2, MAPK10, BCL2L11, MECOM, LPAR6, RARB, E2F2, CALM3, STAT6, RUNX1T1
ssc04659:Th17 cell differentiation	9	MAPK10, FOXS1, SMAD3, STAT6, AHR, FOS, CD3E, RUNX1, TGFBR2
ssc05202:Transcriptional misregulation in cancer	9	ETV7, HHEX, MEIS1, BCL6, KLF3, MLF1, RUNX1T1, RUNX1, TGFBR2
ssc05132:Salmonella infection	9	MAPK10, DYNC2LI1, TCF7L2, ARHGEF26, ELMO1, CASP1, TNFSF10, FOS, STX10
ssc05206:MicroRNAs in cancer	9	EFNA1, BCL2L11, GLS2, DNMT3A, DDIT4, SPRY2, E2F2, EFNA4, FOXP1
ssc04068:FoxO signaling pathway	8	MAPK10, SMAD3, BCL2L11, BCL6, TNFSF10, FBXO32, SOD2, TGFBR2
ssc05225:Hepatocellular carcinoma	8	NQO1, WNT10B, TCF7L2, SMAD3, IGF2, MGST1, E2F2, TGFBR2
ssc04310:Wnt signaling pathway	8	MAPK10, WNT10B, TLE3, TCF7L2, SMAD3, DAAM1, TBL1XR1, PRICKLE2
ssc05166:Human T-cell leukemia virus 1 infection	8	MAPK10, EGR1, SMAD3, TSPO, E2F2, FOS, CD3E, TGFBR2
ssc05161:Hepatitis B	7	MAPK10, SMAD3, E2F2, STAT6, FOS, TLR3, TGFBR2
ssc04360:Axon guidance	7	EFNA1, TRPC6, SEMA3C, SEMA3D, SEMA3B, EFNA4, PLXNA4
ssc05133:Pertussis	6	MAPK10, CASP1, CALM3, C4BPA, FOS
ssc05210:Colorectal cancer	6	MAPK10, TCF7L2, SMAD3, BCL2L11, FOS, TGFBR2
ssc01240:Biosynthesis of cofactors	6	NQO1, PANK1, KYNU, NME3, DHRS3, UGT1A6
ssc04520:Adherens junction	5	LOC110256054, TCF7L2, SMAD3, SSX2IP, TGFBR2
ssc05220:Chronic myeloid leukemia	5	SMAD3, MECOM, E2F2, RUNX1, TGFBR2
ssc02010:ABC transporters	4	ABCC3, ABCG5, ABCC5, ABCA4
ssc00140:Steroid hormone biosynthesis	4	LOC100739741, AKR1C1, AKR1D1, UGT1A6
